# Click Chemistry Derived Hexa-ferrocenylated 1,3,5-Triphenylbenzene for the Detection of Divalent Transition Metal Cations

**DOI:** 10.1021/acsomega.4c04300

**Published:** 2024-09-05

**Authors:** Stanisław Kulczyk, Agata Kowalczyk, Jakub S. Cyniak, Mariola Koszytkowska-Stawińska, Anna M. Nowicka, Artur Kasprzak

**Affiliations:** † Faculty of Chemistry, Warsaw University of Technology, Noakowskiego Street 3, 00-664 Warsaw, Poland; ‡ Faculty of Chemistry, University of Warsaw, Pasteura Street 1, 02-093 Warsaw, Poland

## Abstract

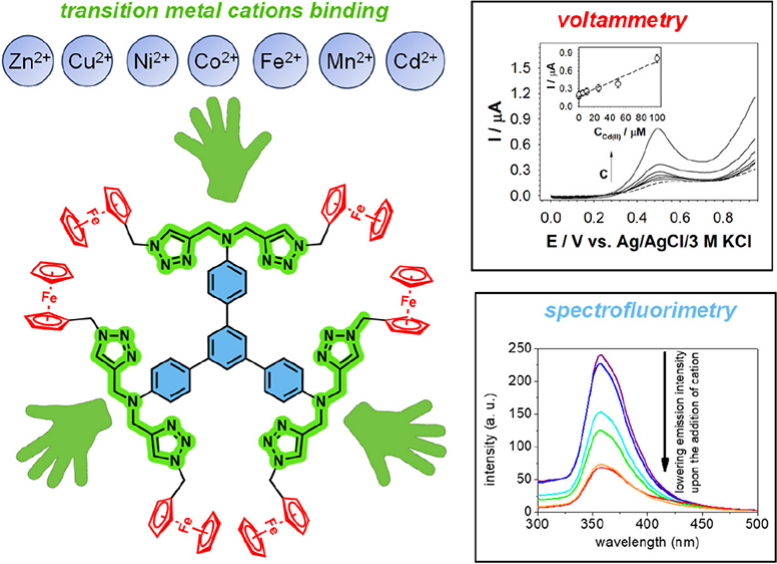

The 1,3-dipolar cycloaddition reaction (click chemistry approach) was employed to create a hexa-ferrocenylated 1,3,5-triphenylbenzene derivative. Leveraging the presence of metal-chelating sites associated with 1,2,3-triazole moieties and 1,4-dinitrogen systems (ethylenediamine-like), as well as tridentate chelating sites (1,4,7-trinitrogen, diethylene triamine-like) systems, the application of this molecule as a chemosensor for divalent transition metal cations was investigated. The interactions were probed voltammetrically and spectrofluorimetrically against seven selected cations: iron(II) (Fe^2+^), cobalt(II) (Co^2+^), nickel(II) (Ni^2+^), copper(II) (Cu^2+^), zinc(II) (Zn^2+^), cadmium(II) (Cd^2+^), and manganese(II) (Mn^2+^). Electrochemical assays revealed good detection properties, with very low limits of detection (LOD), for Co^2+^, Cu^2+^, and Cd^2+^ in aqueous solution (0.03–0.09 μM). Emission spectroscopy experiments demonstrated that the title compound exhibited versatile detection properties in solution, specifically turn-off fluorescence behavior upon the addition of each tested transition metal cation. The systems were characterized by satisfactory Stern–Volmer constant values (10^5^–10^6^ M^–1^) and low LOD, especially for Zn^2+^ and Co^2+^ (at the nanomolar concentration level).

## Introduction

Polyaromatic compounds are important organic molecules used in various applications. Derivatives of 1,3,5-triphenylbenzene are prominent examples in this family. They are commonly used in the creation of organized materials, such as (metal)organic frameworks,^1−3^ dendritic molecules^4−7^ or (metal)organic cages.^8−11^ From a synthetic viewpoint, numerous methods exist for synthesizing 1,3,5-triphenylbenzene congeners with various functional groups. Typically, derivatization involves installing three functional groups at all three 4-positions of the outer phenyl rings of 1,3,5-triphenylbenzene. This approach enables tuning the structure of this *C*_3_-symmetric polyaromatic backbone for specific applications.

The use of π-conjugated polyaromatics in the design of molecular chemosensors for ions has been intensively studied in recent years. Molecular chemosensors are organic molecules that recognize analytes through noncovalent interactions and produce a detectable signal, such as an optical or electrochemical response.^12^ Due to the π-conjugated structure of 1,3,5-triphenylbenzene, its derivatives can serve as optical chemosensors.^13,14^ The analytical response can be tracked using spectrofluorimetry or absorption spectroscopy. Such applications of 1,3,5-triphenylbenzene-based optical chemosensors have been demonstrated in the recognition of various ions.^14−19^ On the other hand, installing redox-active moieties onto a polyaromatic skeleton opens avenues for designing electrochemical chemosensors. For this type of chemosensor, a detectable analytical response is provided by a redox probe. Typically, the linker between a polyaromatic skeleton and a redox moiety is included in the interactions between chemosensors and analytes. Ferrocene (Fc) is one of the best choices for designing electrochemical chemosensors due to its one-electron, reversible, and quantitative oxidation to ferrocenium cation.^20−22^ Additionally, many commercially available or easily synthesized, air-stable Fc derivatives bear synthetically useful moieties. Over the years, numerous reports have been published on merging the chemistry of Fc with π-conjugated polyaromatics,^23−27^ including the design of electrochemical chemosensors for ions.^28−33^ Regarding Fc-decorated 1,3,5-triphenylbenzene or its congeners, most reports focus on synthesis design, photophysical properties, and basic electrochemical studies.^34−38^ However, to the best of our knowledge, the application of ferrocenylated 1,3,5-triphenylbenzenes as chemosensors for ions or metal-complexing agents is limited, with only a few essential examples.^39−41^

**Figure 1:**
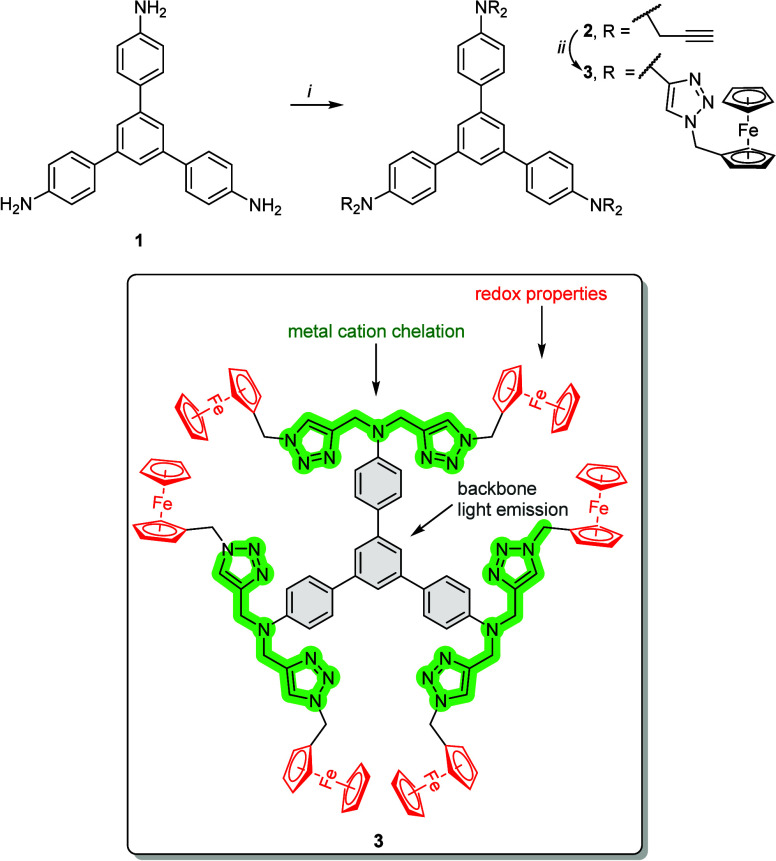
Synthesis of compounds **2** and **3** (reagents and reaction conditions: *i*. K_2_CO_3_, propargyl bromide, acetonitrile, 48 h, reflux, 97% yield; *ii*. (ferrocenylmethyl)azide, (CH_3_CO_2_)_2_Cu, sodium ascorbate, H_2_O, *t*-butanol, room temperature, 5 days, 42% yield). Structure of target compound **3** with the structural motifs marked with colors is also presented.

In pursuit of expanding the library of redox-active 1,3,5-triphenylbenzene-based chemosensors, we report the design of a click chemistry-derived hexa-ferrocenylated 1,3,5-triphenylbenzene (compound **3**) and its application as a chemosensor molecule dedicated to the detection of transition metal cations. The structure of compound **3** is presented in Figure 1. The 1,3,5-triphenylbenzene skeleton (marked in gray in Figure 1) was used as a C_3_-symmetric backbone, enabling structural expansion of the molecule. This motif also provided light emission properties to compound **3**. The redox properties of compound **3** were provided by the presence of six Fc units (marked in red in Figure 1). The metal cation chelation properties of compound **3** were anticipated due to the presence of 1,2,3-triazole skeletons and 1,4-dinitrogen (ethylenediamine-like) systems (marked in green in Figure 1). The molecular design and binding mode were supported by density functional theory (DFT) computational investigations. The 1,2,3-triazole skeletons additionally contributed to the light emission properties of compound **3**. The recognition properties of compound **3** were tested voltammetrically and spectrofluorimetrically against selected divalent transition metal cations, namely iron(II) (Fe^2+^), cobalt(II) (Co^2+^), nickel(II) (Ni^2+^), copper(II) (Cu^2+^), zinc(II) (Zn^2+^), cadmium(II) (Cd^2+^), and manganese(II) (Mn^2+^). These divalent transition metal cations were selected for our receptor studies due to the demonstrated binding features of 1,2,3-triazoles^42^ and ethylenediamine-like systems.^43^

## Results and Discussion

Refer to Supporting Information for experimental details on the synthesis of compounds **2** and **3** and their characterization data (1D and pseudo 2D DOSY NMR, qNMR, HRMS). In brief, the synthesis of compound **3** involved two steps (Figure 1). First, 1,3,5-tris(4-aminophenyl)benzene **1**(44) was propargylated using an excess of propargyl bromide in acetonitrile in the presence of potassium carbonate. The propargyl intermediate **2** was obtained in a very high yield (97%). Interestingly, the crude compound **2** did not require purification before its conversion into compound **3**. The target compound **3** was obtained in a yield of 42% via the copper-catalyzed 1,3-dipolar cycloaddition reaction (click chemistry approach) between hexa-propargylated 1,3,5-triphenylbenzene **2** and (ferrocenylmethyl)azide.^45^ The reaction was performed in a *tert*-butanol–H_2_O solvent system using copper(II) acetate and sodium ascorbate. The synthesis of compound **3** was chromatography-free. Compound **3** was obtained as a brown amorphous solid through filtration and washing with solvents. As determined by ^1^H qNMR analysis (Figure S4), this convenient purification method provided compound **3** with 98% purity.

**Figure 2:**
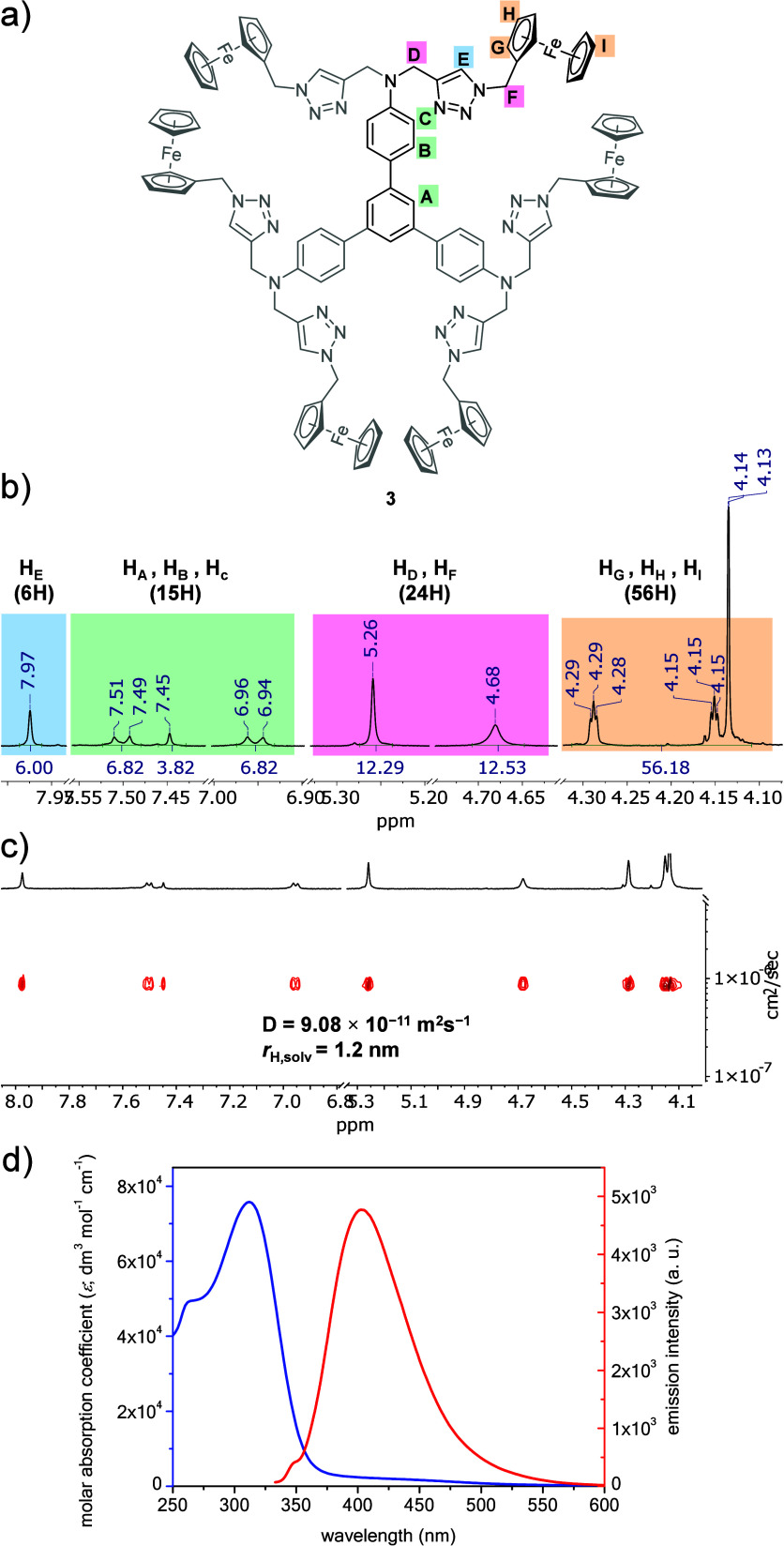
Representative characterization data for compound **3**: (**a**) structure of **3** with atom labels for ^1^H NMR analysis, inset of the (**b**) ^1^H NMR (DMSO-*d*_6_, 500 MHz) and (**c**) ^1^H DOSY NMR spectra of **3** (DMSO-*d*_6_, 500 MHz), (**d**) UV–vis (blue curve) and emission (λ_ex_ = 312 nm; red curve) spectra of **3** in DMSO (2 × 10^–5^ M).

The ^1^H NMR spectrum of compound **3** in DMSO-*d*_6_ comprised nine groups of signals (Figure 2b). The singlet at 7.97 ppm was attributed to the H_E_ protons of the 1,2,3-triazole moiety (see atom labels in Figure 2a). Multiplets at 7.51–7.49 ppm (H_B_) and 6.96–6.94 ppm (H_C_), as well as a singlet at 7.45 ppm (H_A_), were attributed to the protons within the 1,3,5-triphenylbenzene skeleton. Two singlets at 5.26 and 4.68 ppm were assigned to the methylene moieties (H_D_ and H_F_ protons). The remaining signals between 4.29 and 4.13 ppm were attributed to the protons from the substituted (H_G_, H_H_) and unsubstituted (H_I_) cyclopentadienyl rings of Fc. The ^1^H DOSY NMR experiment (Figure 2c) confirmed that the sample of compound **3** is composed of a single molecule with a diffusion coefficient (D) of 9.08 × 10^–11^ m^2^ s^–1^ and an approximate hydrodynamic radius (*r*_H,solv_) of about 1.2 nm (*r*_H,solv_ was calculated using the Stokes–Einstein equation^46,47^).

The UV–vis spectrum of compound **3** in DMSO featured two major absorption maxima (λ_abs_) located at 264 and 312 nm (Figure 2d, blue curve). These λ_abs_ can be attributed to π–π* transitions^48^ and are characterized by molar absorption coefficient (*ε*) values of 4.95 × 10^4^ dm^3^ mol^–1^ cm^–1^ and 7.45 × 10^4^ dm^3^ mol^–1^ cm^–1^ for λ_abs_ at 264 and 312 nm, respectively. The emission maximum (λ_em_) for compound **3** was found at approximately 402 nm (Figure 2d, red curve).

**Figure 3:**
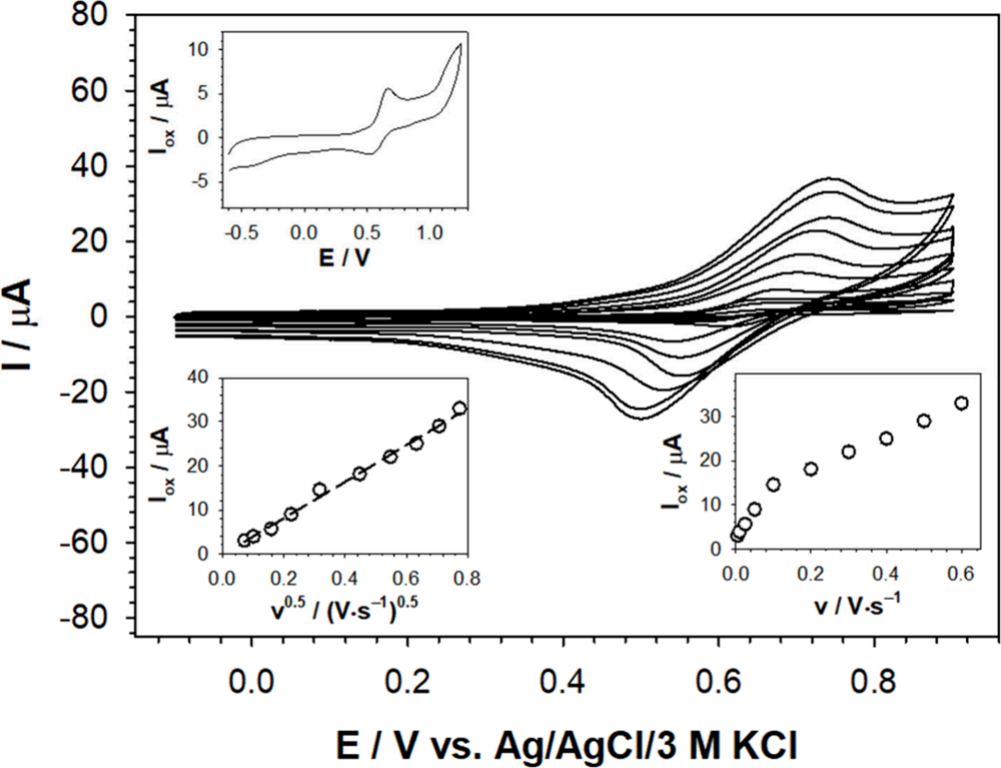
Cyclic voltammograms (CVs) of compound **3** recorded in the mixture of DMSO–DCM (*v*/*v*; 1:3) with the addition of 50 mM tetrabutylammonium hexafluorophosphate (TBAPF_6_). Top inset: CV voltammogram for scan rate 0.05 V s^–1^. Bottom insets: Dependencies of anodic peak currents vs square root of scan rate (left) and scan rate (right). Experimental conditions: *C*_**3**_ = 0.17 mM, *C*_TBAPF6_ = 50 mM, *T* = 21 °C.

Compound **3** contained six electrochemically active Fc units. Generally, the length of the linker between Fc units determines whether these redox centers communicate with each other. To investigate the redox behavior of compound **3**, a cyclic voltammetric (CV) curve was recorded (see the top inset in Figure 3). The recorded CV curve showed one pair of peaks: an oxidation peak at approximately 0.67 V and a reduction peak at approximately 0.54 V. This indicated a lack of electronic communication between the Fc units during the exchange of an electron with the electrode surface. The presence of both cathodic and anodic signals confirmed the reversible nature of the electrode process for compound **3**.

To determine the nature of the recorded current signal, CV curves were recorded at different scan rates ranging from 0.005 to 0.6 V s^–1^ (Figure 3). Based on the recorded CV curves, the dependencies of the oxidation peak current of the Fc units on the scan rate and the square root of the scan rate were plotted. For compound **3**, the relationship *I*_p_ = f(*v*^0.5^) was linear, whereas the relationship *I*_p_ = f(*v*) was nonlinear, as shown in the insets in Figure 3. Thus, the electrode process of compound **3** was diffusive, indicating that the limiting step of the electrode process was the diffusion of the electroactive substance to the electrode surface. This behavior for compound **3** confirmed the linear nature of the relationship presented in the bottom left inset in Figure 3.

Having electrochemically characterized compound **3**, its application as the crucial ingredient of the receptor layer of a voltammetric sensor dedicated to the detection of divalent transition metal cations was investigated. Seven cations were selected for these studies, namely Fe^2+^, Co^2+^, Ni^2+^, Cu^2+^, Zn^2+^, Cd^2+^, and Mn^2+^. First, to prepare the receptor layer, the surface of a glassy carbon electrode was modified with compound **3** by applying a 7 μL droplet of a solution (1.7 mM of compound **3** in a mixture of DMSO–DCM (*v*/*v*; 1:3) with 50 mM tetrabutylammonium hexafluorophosphate (TBAPF_6_) and 5% Nafion) and leaving it to dry. The as-prepared electrode was immersed in a 100 mM aqueous solution of TBAPF_4_, into which the cation was then introduced at the specified concentration.

**Figure 4:**
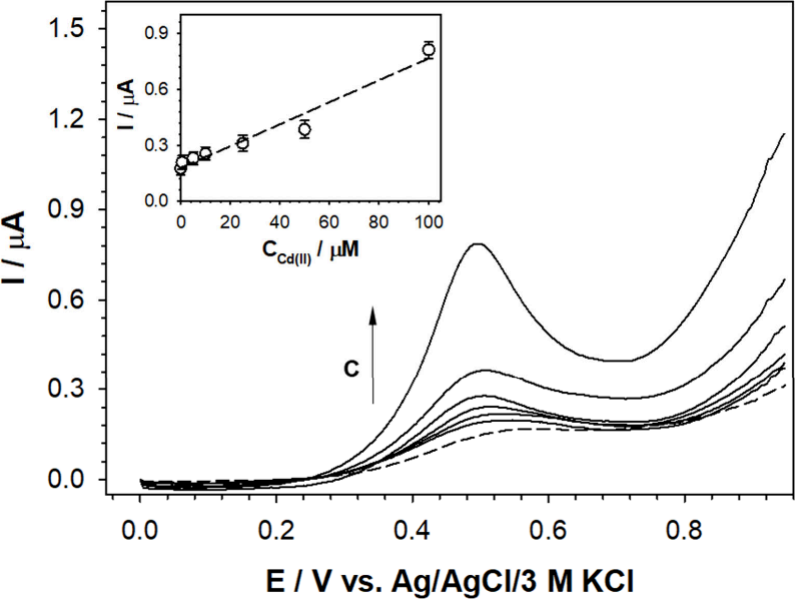
DP voltammograms of the receptor (GC/compound **3**-Nafion-TBAPF_6_) recorded in 100 mM TBABF_4_ aqueous solution (dashed lines) with different addition of Cd^2+^ as the representative analyte (solid lines). Insets: Dependencies of anodic peak currents vs Cd^2+^ concentration. Experimental conditions: *C*_**3**_ = 1.7 mM, *T* = 21 °C, modulation time: 0.002 s, interval time: 0.1 s, modulation amplitude: 0.04995 V.

The recorded differential pulse (DP) voltammograms for Cd^2+^ as the representative analyte are shown in Figure 4 (refer to SI, Section S4, for DP voltammograms for other analytes). A linear dependence of the current signal as a function of transition metal ion concentration was observed only for Cu^2+^ and Cd^2+^. In the presence of Co^2+^ in solution, an increase in the current signal was also observed, but in a much narrower range of cation concentrations. For the other tested cations, the opposite effect was observed; as the concentration of the cation in solution increased, the recorded current signals decreased in intensity. Therefore, it can be concluded that for the constructed voltammetric sensor and under these conditions, only Cu^2+^, Cd^2+^, and Co^2+^ cations formed stable complexes with compound **3** in the receptor layer.

Based on the obtained voltammetric dependencies, the analytical range of work, limit of detection (LOD), and limit of quantification (LOQ) values were determined for Cu^2+^, Cd^2+^, and Co^2+^. The LOD and LOQ were calculated according to the following eqs 1, 2):
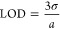
1
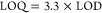
2where σ is the standard deviation of the response observed for the lowest measurable cation concentration and *a* is the slope of the calibration curve. The linear correlation equations and analytical parameters are given in Table 1. The calculated LOD values were low (<0.1 μM). The lowest LOD value was found for Cu^2+^ (0.03 μM), whereas the highest was for Co^2+^ (0.09 μM). The lowest LOD for Cu^2+^ might be associated with its highest acidity, resulting from the lowest ionic radius of Cu^2+^ (73 pm) among the three analytes tested (the ionic radius for Co^2+^ and Cd^2+^ is ≥79 pm).^49^

**Figure 5:**
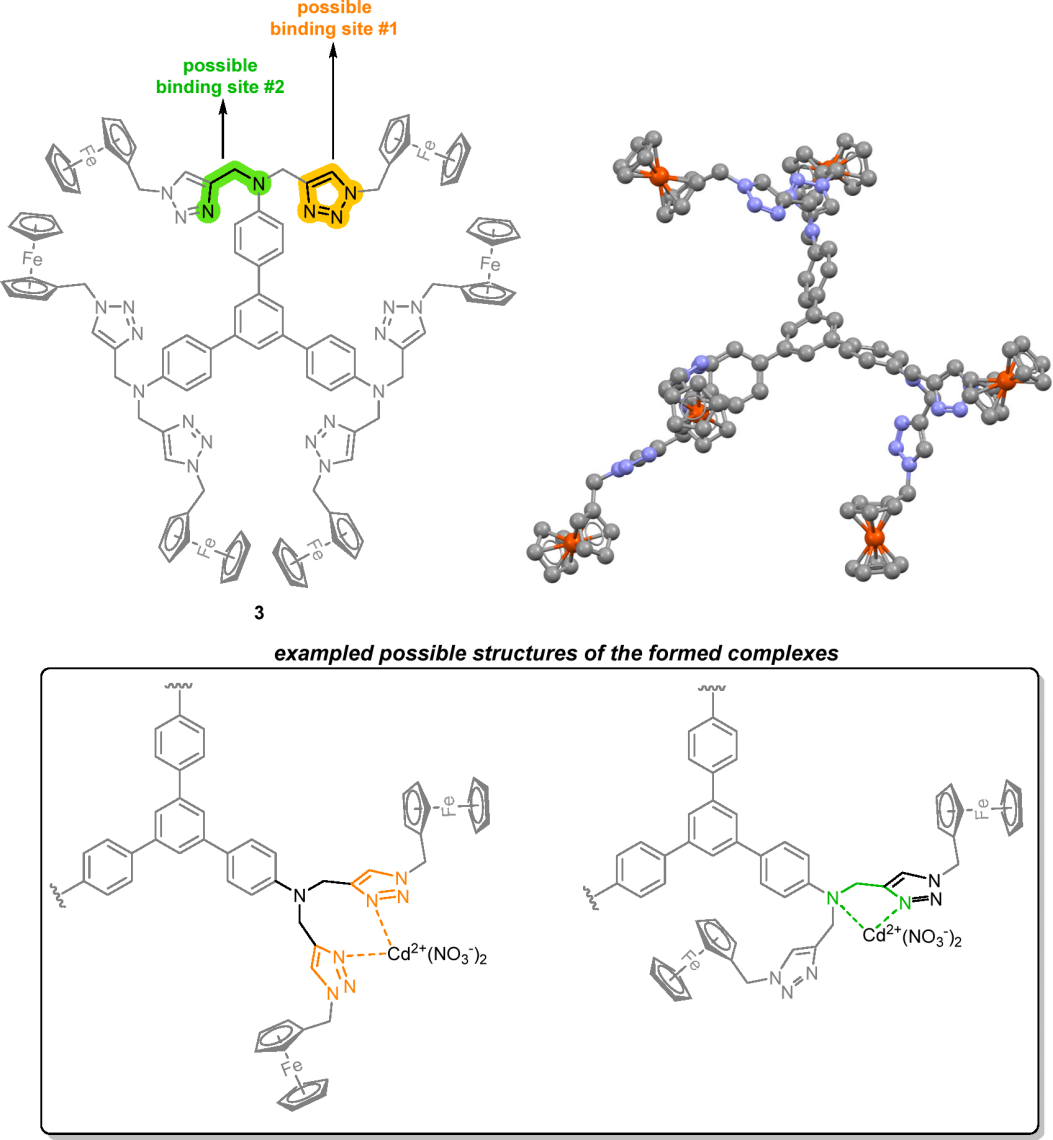
Possible binding sites for noncovalent interactions between compound **3** and cationic species together with exampled possible structures of the formed complexes (water molecules are not included for the clarity of the image; structures are presented with cadmium(II) as the representative cation). The PM6-optimized structure of **3** is also presented (hydrogen atoms are omitted for clarity).

Notably, the LOD for the detection of Cu^2+^ in aqueous solution using the 3-containing voltammetric sensor was lower than that of reported ferrocenylated molecular Cu^2+^ chemosensors, what is beneficial in terms of sensors' application potential (see comparison data in Table 2). We believe this improvement resulted from the presence of several possible chelating sites in the structure of compound **3**, related to the presence of 1,2,3-triazole skeletons and 1,4-dinitrogen systems (ethylenediamine-like systems), as illustrated in Figure 5 and discussed further. Achieving effective detection of Co^2+^ and Cr^2+^ with the 3-containing voltammetric sensor is also significant, given that reports on the detection of these transition metal cations with ferrocenylated molecular chemosensors are sparse.^50−53^

**Table 1:** Values of the Analytical Parameters for the Voltammetric Sensor Containing Compound **3** in the Receptor Layer

**Entry**	**Analyte**	**Regression equation**	***r***	**Linear range of work [μM]**	**LOD [μM]**	**LOQ [μM]**
1	Cu^2+^		0.993	5–100	0.03	0.099
2	Cd^2+^		0.961	5–100	0.06	0.165
3	Co^2+^		0.902	5–25	0.09	0.297

**Table 2:** Comparison of the LOD Values for the Voltammetric Detection of Cu^2+^ by **3**-Containing Sensor and Reported Ferrocenylated Molecular Chemosensors

**Entry**	**Chemosensor molecule**	**LOD [μM]**	**ref**
**1**	compound 3	**0.03**	**This work**
2	Fc-chalcone conjugates	0.79–10.00	(54−56)
3	Fc-rhodamine B conjugates	2.00–6.85	(57, 58)
4	Fc-rhodamine 6G conjugate	3.00	(29)
5	Fc-containing azine	17.80	(59)
6	Selenium-doped Fc-anthracene conjugates	52.60	(60)

Taking into account the structure of compound **3**, which consists of six moieties attached to one 1,3,5-triphenylbenzene backbone, at least two possible binding sites can be considered (see Figure 5). The first possible binding site (marked orange in Figure 5) includes the 1,2,3-triazole moieties, whose properties for interacting with transition metal cations have been demonstrated in the literature.^42^ This binding site could be potentially considered predominant in the studied systems with compound **3**, due to the high feasibility of 1,2,3-triazole skeletons to interact with metallic species. The second possible binding site (marked green in Figure 5) is related to the presence of 1,4-dinitrogen (ethylenediamine-like) moieties in compound **3**. This binding site could be considered possible due to the common presence of ethylenediamine-like moieties in metal chelating systems.^43^

Figure 5 also presents possible, exampled molecular arrangements of the dynamically formed complexes for cobalt(II) (Co^2+^) as a representative analyte. Due to the noted lack of electronic communication between the Fc units observed in the CV experiments (Figure 3 and related discussion), no cooperativity effect of the six redox (Fc) centers in the voltammetric recognition process was considered. However, the cooperative effect of the two 1,2,3-triazole motifs attached to the same “arm” part of molecule **3** is possible. Additionally, the simultaneous inclusion of both 1,2,3-triazole-based and 1,4-dinitrogen-based binding modes in one complex is also likely. These effects could be considered possible due to the flexibility of molecule **3**, which is related to the presence of the 1,3,5-triphenylbenzene skeleton with twisted phenylene rings, as well as the C(sp^3^) carbon atoms (methylene bridges) derived from propargylation and click chemistry reactions. To visualize these considerations, the optimization of the structure of compound **3** was performed using Gaussian09W software^61^ (since compound **3** contained 234 atoms in total, including 6 iron(II) atoms, semiempirical PM6 method^62−64^ was selected). The optimized structure of compound **3** (Figure 5) well visualizes the noted considerations: the phenylene rings in the 1,3,5-triphenylbenzene backbone are twisted, and the methylene bridges provide flexibility to the introduced moieties attached to the 1,3,5-triphenylbenzene skeleton.

**Figure 6:**
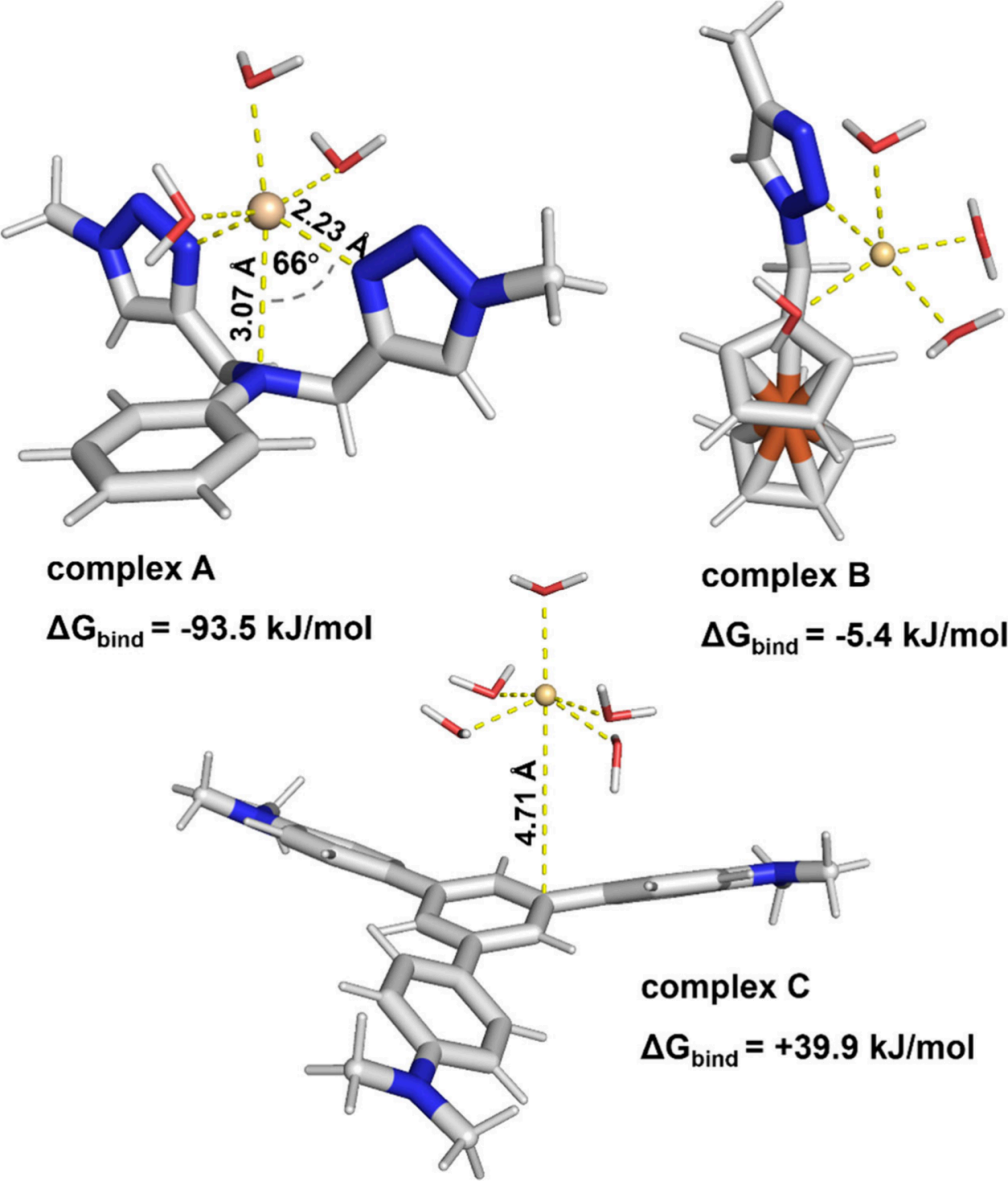
DFT-predicted structures and binding energies of relevant fragments of **3** with Cd^2+^ cation and water molecules.

To further investigate the possible molecular arrangements, as well as to estimate interaction energies, density functional theory (DFT) computations (geometry optimizations and frequency calculations) were performed with Orca program using BP86 method together with polarizable continuum model (PCM).^65−67^ The computational details were included in the SI, section **S7**. Three possible complexes of relevant fragments of **3** with Cd^2+^ cation and water were considered. The DFT optimized structures of the considered complexes and their binding energies were shown on Figure 6: a) with Cd^2+^ bound to the 1,4-dinitrogen system, (complex A), b) with Cd^2+^ bound to one 1,2,3-triazole nitrogen atom and cyclopentadienyl ring of the ferrocene moiety (complex B), c) with Cd^2+^ bound to the triphenylene core of the molecule (complex C).

Out of the three considered complexes, the nitrogen-oriented complex A had the lowest binding energy (−93.5 kJ/mol). This finding suggested that the formation of this complex in the solution was favorable. On the other hand, the formation of non-nitrogen-oriented complexes was found to be only slightly favorable (−5.4 kJ mol^–1^, complex B) or unfavorable (+39.9 kJ mol^–1^, complex C).

In the nitrogen-oriented complex A, Cd^2+^ was coordinated to two triazole nitrogen atoms. In addition, the nonplanar configuration of the aniline-like nitrogen atom suggested that this atom also interacts with Cd^2+^. Therefore, the cation was coordinated by a 1,4,7-trinitrogen (diethylenetriamine-like) system, rather than simply by two 1,2,3-triazole moieties or by a single 1,4 ethylenediamine system. The distance of Cd^2+^ to triazole nitrogen atoms was as low as 2.23 Å, which suggested a strong interaction. It was shorter than its distance to the aniline-like nitrogen atom (3.07 Å) and shorter than its average distance to water oxygen atoms (2.39 Å). On the other hand, in carbon-oriented complex C the distance between the aromatic system and Cd^2+^ was as large as 4.71 Å, which suggested a very weak interaction. In carbon oriented complex B the large distance and angle between Cd^2+^ and cyclopentadienyl ring indicated that there is no interaction between them.

The above conclusions demonstrated the importance of the presence of a 1,4,7-trinitrogen (diethylenetriamine-like) skeleton in the structure of **3**. The presence of this skeleton provided **3** with its tridentate ligand character.

**Figure 7:**
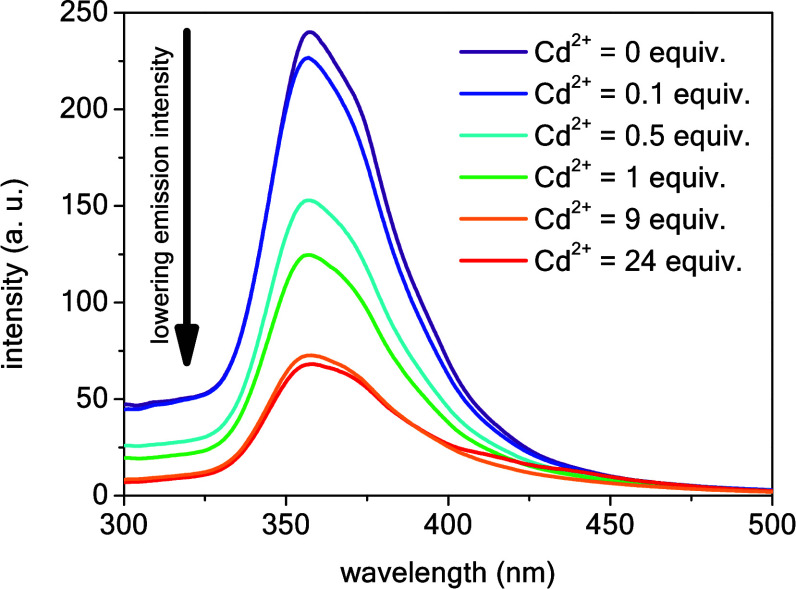
Changes in the emission spectra of **3** in the presence of Cd^2+^ as the representative analyte (*C*_**3**_ = 2 × 10^–7^ M, DMSO–H_2_O 1:1 *v*/*v*, λ_ex_ = 270 nm).

Finally, to further investigate the interactions between compound **3** and the studied transition metal cations experimentally, binding experiments in solution were performed using spectrofluorimetry. These assays were conducted in a DMSO:H_2_O (1:1 *v*/*v*) solvent system to ensure the solubility of both the chemosensor (compound **3**) and the analyte (transition metal cation) in the solution. The concentration of compound **3** in each sample was 2 × 10^–7^ M to avoid any solubility or precipitation issues during the assays, taking into account its high molar mass and relatively poor solubility in DMSO. The emission intensity for compound **3** (λ_em_ = 356 nm) systematically decreased (turn-off fluorescence behavior) upon the addition of increasing molar equivalents of a cation, as shown in Figure 7 for the spectra of compound **3** in the presence of Cd^2+^ as the representative analyte (refer to **Figure S9** in the SI for a summary of data for different cations). This indicated that contrary to the electrochemical assays where complex formation was observed only with Cu^2+^, Cd^2+^, and Co^2+^, compound **3** exhibited the versatile property of recognizing each tested transition metal cation in solution. We believe this observation results from the differences between these techniques. For the voltammetric sensor, the chemosensor (compound **3**) is permanently adsorbed on the electrode surface. To ensure that the adsorption process does not restrict the orientation of the receptor in the layer and permanently binds it to the electrode surface, a receptor cross-linking process using Nafion was used. It should be noted that the cross-linking process itself can lead to steric hindrance. In contrast, in solution (emission spectroscopy measurements), both the chemosensor and analyte molecules are dissolved in the same liquid medium. This results in a more dynamic system, which enables conformational changes of the receptor toward binding the analyte.

Stern–Volmer plots were used to evaluate the detection ability of compound **3** toward the investigated divalent transition metal cations (refer to SI, Section S5, for the plots).^68−71^ The Stern–Volmer constant (*K*_SV_) values for the systems were satisfactory, ranging from 0.09 × 10^6^ M^–1^ to 0.93 × 10^6^ M^–1^, (see Table 3). The highest *K*_SV_ value was found for Ni^2+^ (0.93 × 10^6^ M^–1^). Possible stoichiometry of the dynamically formed complexes in solution was estimated spectrofluorimetrically with the continuous variation method (Job’s plot method). Stoichiometry (cation:receptor) was 1:1 for Co^2+^, Cu^2+^, Fe^2+^, and Ni^2+^, whereas, 3:1 for Zn^2+^ and Cd^2+^, and 1:3 for Mn^2+^ (refer to SI, Section S6, for the Job’s plots). We believe the differences between these predicted stoichiometries in solution might result from different coordination number preference for tested cations. For example, Co^2+^, Cu^2+^, Fe^2+^, and Ni^2+^ could be considered to prefer coordination number 6 in complexes, whereas Zn^2+^ and Cd^2+^ feature coordination numbers 4 and 6, depending on the ligand structure.

**Table 3:** Summary of *K*_SV_ and LOD Values for the Systems Comprising **3** and Given Transition Metal Cation (Values Estimated from Emission Spectroscopy)

**Entry**	**Analyte**	***K***_**SV**_**[M**^**–1**^**]**	**LOD [μM]**
1	Ni^2+^	0.93 × 10^6^	0.20
2	Zn^2+^	0.70 × 10^6^	0.08
3	Cd^2+^	0.56 × 10^6^	0.30
4	Mn^2+^	0.22 × 10^6^	0.78
5	Fe^2+^	0.21 × 10^6^	0.55
6	Co^2+^	0.11 × 10^6^	0.11
7	Cu^2+^	0.09 × 10^6^	4.20

In general, LOD values for the systems were low, not higher than 0.8 μM, with the only exception being Cu^2+^ (4.2 μM, see Table 3; refer to SI, Section S5, for the plots). The highest LOD for Cu^2+^ might be associated with the lowest *K*_SV_ for this analyte (0.09 × 10^6^ M^–1^). The most satisfactory LOD values were found for Zn^2+^ (0.08 μM) and Co^2+^ (0.11 μM), suggesting the possibility of fluorescent detection of these cations with chemoreceptor **3** even at the nanomolar concentration level. The LOD values estimated from voltammetric and spectrofluorimetric data cannot be directly compared due to the differences between these techniques (solution versus receptor layer). However, it can be observed that the LOD values for Co^2+^ estimated from voltammetric (0.09 μM) and spectrofluorimetric (0.11 μM) studies were similar. Additionally, the LOD values for Zn^2+^ and Co^2+^ estimated from spectrofluorimetric studies were at a similar level to the LOD values for Co^2+^, Cd^2+^, and Cu^2+^ estimated from voltammetric assays.

## Conclusions

In conclusion, we demonstrated that the introduction of six ferrocene moieties to the 1,3,5-triphenylbenzene framework can be achieved via a click chemistry approach using a hexa-propargylated 1,3,5-triphenylbenzene derivative and (ferrocenylmethyl)azide as starting materials. Thanks to the presence of a tridentate chelating site related to the presence of 1,4,7-trinitrogen (diethylene triamine-like) system, the target molecule featured the property of recognizing transition metal cations, as supported with DFT computational investigations. We showed that the title compound could be used as both an electrochemical and optical chemosensor. The constructed voltammetric sensor effectively detected Co^2+^, Cu^2+^, and Cd^2+^ in aqueous solution, elucidated by very low LOD values ranging from 0.03 to 0.09 μM. The proposed sensor showed an improved LOD for Cu^2+^ voltammetric detection compared to reported devices comprising other ferrocenylated polyaromatics. Spectrofluorimetric experiments in solution revealed that the title compound exhibited emission quenching (turn-off fluorescence behavior) upon the addition of each tested cation, namely Fe^2+^, Co^2+^, Ni^2+^, Cu^2+^, Zn^2+^, Cd^2+^, or Mn^2+^. The interactions were characterized by satisfactory Stern–Volmer constant (*K*_SV_) values at the level of 10^5^–10^6^ M^–1^. Especially low LOD values were found for Zn^2+^ (0.08 μM) and Co^2+^ (0.11 μM), revealing the possibility of fluorescent detection of these cations with the title compound even at the nanomolar concentration level. This work not only demonstrates the possibilities of synthesizing structurally expanded multiferrocenylated polyaromatics but also suggests the benefits of installing several possible binding sites in such derivatives to improve their ion-recognition properties.

## Supporting Information Available

Materials and methods, experimental section, compound characterization data, details and additional data on electrochemical studies and cation binding experiments, DFT computational details (PDF)
