# Supplementary material for: Click Chemistry Derived Hexa-ferrocenylated 1,3,5-Triphenylbenzene for the Detection of Divalent Transition Metal Cations
Source: ACS Omega. 2024 Sep 5;9(37):38658–67. doi: 10.1021/acsomega.4c04300 (PMC11411552; doi:10.1021/acsomega.4c04300)
Supplement: Supplementary file 1 — ao4c04300_si_001.pdf [file ao4c04300_si_001.pdf]

SUPPORTING INFORMATION (SI) FOR

Click chemistry derived hexa-ferrocenylated 1,3,5-triphenylbenzene for the detection of divalent transition metal cations

Stanisław Kulczyk,[a,] Agata Kowalczyk,[b] Jakub S. Cyniak,[a] Mariola Koszytkowska-Stawińska,[a]* Anna M. Nowicka,[b] Artur Kasprzak[a]*

[a] Faculty of Chemistry, Warsaw University of Technology, Noakowskiego Str. 3, 00-664 Warsaw, Poland

* Corresponding authors e-mails: mariola.koszytkowska@pw.edu.pl (M.K.-S.), artur.kasprzak@pw.edu.pl (A.K.)

[b] Faculty of Chemistry, University of Warsaw, Pasteura Str. 1, 02-093 Warsaw, Poland

Table of contents

S1. Materials and methods S2
S2. Synthesis S4
S3. Spectral characterization data for 2-3 .... S5
S4. Electrochemical analyzes S10
S5. Stern-Volmer plots for the interactions between 3 and metal cations S11
S6. Job's plots for the interactions between 3 and metal cations S14
S7. DFT calculations methodology S15
S8. Supporting references S16

S1. Materials and methods

Chemical reagents and solvents were commercially purchased and purified according to the standard methods, if necessary. 1,3,5-tris(4-aminophenyl)benzene (**1**)[1] and (ferrocenylmethyl)azide[2] were synthesized following the literature procedures.

NMR experiments were carried out using a Varian VNMRS 500 MHz spectrometer (^{1}H at 500 MHz, ^{13}C{^{1}H} NMR at 126 MHz) equipped with a multinuclear z-gradient inverse probe head. The spectra were recorded at 25 °C and standard 5 mm NMR tubes were used. ^{1}H and ^{13}C chemical shifts (δ) were reported in parts per million (ppm) relative to the solvent signal, *i.e.*, Chloroform-d: δ_H (residual chloroform) 7.26 ppm, δ_C (residual chloroform) 77.16 ppm; DMSO-d_6: δ_H (residual DMSO) 2.50 ppm. ^{1}H DOSY (Diffusion Ordered SpectroscopY) NMR experiments were performed using a stimulated echo sequence incorporating bipolar gradient pulses[3] and with convection compensation.[4] The gradient strength was logarithmically incremented in 15 steps from 25% up to 95% of the maximum gradient strength. NMR spectra were analyzed with the MestReNova v12.0 software (Mestrelab Research S.L). The hydrodynamic radius from ^{1}H DOSY NMR experiment was estimated using the unmodified Stokes-Einstein equation[5,6]:

$$r_{H,solv} = \frac{k_B T}{6\pi\eta D}$$

where D is the measured diffusion coefficient for compound **3** ($9.08\cdot10^{-11}$ m^{2}s^{-1}), k_B is the Boltzmann constant ($1.3806485\cdot10^{-23}$ kg·s^{-2}K^{-1}), T is the temperature for the ^{1}H DOSY NMR spectrum acquisition (298 K), $r_{H,solv}$ is the calculated hydrodynamic radius of compound **3** (*ca.* 1.2 nm), η is the viscosity of the solvent (DMSO) at temperature T (0.001991 kg·m^{-1}s^{-1}).

APCI-HRMS (q-TOF) measurements were recorded using Synapt G2-S HDMS mass spectrometer (*Waters*) equipped with the atmospheric-pressure chemical ionization (APCI) ion source and quadrupole-Time-of-Flight (q-TOF) mass analyzer. Methanol (Honeywell, HPLC-MS Chromasolv, purity ≥ 99.9%) was used as a solvent and mobile phase with the flow rate of 100 μl/min. Sample was dissolved and injected directly into the APCI source. Injection volume was 1 μl. The measurements were recorded in the positive and negative ion modes with the resolving power of TOF analyzer 20 000 FWHM. The lock-spray spectrum of Leucine-enkephalin was generated by the lock-spray source and the correction was performed for recorded mass spectra in the mass range of m/z = 50 – 1200. The exact mass measurements were performed within 3 mDa mass error. Nitrogen was used as the desolvation and cone gas, and their flow values were set to 600 L/h and 100 L/h, respectively. Nebulizer gas pressure was set to 5.0 bar. Source and probe temperatures were set to 120°C and 550°C, respectively. Corona current was set to 13.0 μA, sampling cone voltage and source offset were set to 40 V. The instrument was controlled and data were processed using the MassLynx V4.1 software package (*Waters*).

ESI-HRMS (TOF) measurements were performed with a Q-Exactive ThermoScientific spectrometer.

UV-vis measurements were performed with a WVR UV-1600PC spectrometer, with the spectral resolution of 2 cm^{-1}. For the UV-Vis measurements, the wavelengths for the absorption maxima λ_{max} were reported in nm.

Emission spectra were recorded with a HITACHI F-7100 FL spectrometer, parameters, scan speed: 1200 nm/min, delay: 0.0 s, EX slit: 5.0 nm, EM slit: 5.0 nm, PMT voltage: 400 V. The wavelengths for the emission maxima (λ_{em}) were reported in nm.

Electrochemistry. **Materials:** Dichloromethane (DCM), dimethyl sulfoxide (DMSO), tetrabutylammonium hexafluorophosphate ($TBAPF_6$), tetrabutylammonium tetrafluoroborate ($TBAPF_4$), copper(II) nitrate pentahydrate ($Cu(NO_3)_2{\cdot}5H_2O$), cadmium nitrate tetrahydrate ($Cd(NO_3)_2{\cdot}4H_2O$), cobalt(II) nitrate hexahydrate ($Co(NO_3)_2{\cdot}6H_2O$), iron(II) nitrate heptahydrate, ($Fe(NO_3)_2{\cdot}7H_2O$), manganese(II) nitrate tetrahydrate ($Mn(NO_3)_2{\cdot}4H_2O$), nickel(II) nitrate hexahydrate ($Ni(NO_3)_2{\cdot}6H_2O$), zinc nitrate hexahydrate ($Zn(NO_3)_2{\cdot}6H_2O$), and nafion were purchased from Merck and used as received. **Voltammetric measurements**. The measurements were performed using cyclic voltammetry (CV) and differential pulse voltammetry (DPV) techniques with a potentiostat Autolab PGSTAT 12, in a three-electrode system consisting of: (*i*) working electrode - glassy carbon disc electrode ($\phi = 3.0$ mm), (*ii*) reference electrode - Ag/AgCl/3 M KCl and (*iii*) auxiliary electrode - platinum plate with an area of at least 1 cm^2. Measurements were carried out both in aqueous medium: distilled water with the addition of 100 mM $TBAPF_4$, and non-aqueous medium: a mixture of DMSO:DCM (*vol* : *vol*; 1:3) with the addition of 50 mM $TBAPF_6$. Before the measurements, the surface of the working electrode was cleaned on a polishing wet pad with Al_2O_3 ($\phi = 1.0$ μm). Alumina residues were removed with an intensive stream of ultra-pure water (Hydrolab, conductivity ~0.056 $\mu S{\cdot}cm^{-1}$) perpendicular to the electrode surface.

Cation binding experiments between compound **3** (chemosensor) and cations (analytes; Mn^{2+}, Cd^{2+}, Ni^{2+}, Zn^{2+}, Co^{2+}, Fe^{2+}, Cu^{2+}) were performed employing the emission spectra measurements. Cations were introduced in the form of their corresponding salts: $MnSO_4{\cdot}H_2O$, $Cd(NO_3)_2{\cdot}6H_2O$, $Ni(NO_3)_2{\cdot}6H_2O$, $Zn(NO_3)_2{\cdot}6H_2O$, $Co(NO_3)2{\cdot}_6H_2O$, $Fe(SO_4)_2{\cdot}7H_2O$ and $CuSO_4{\cdot}5H_2O$. The experiments were performed in the DMSO/H_2O = 1:1 *v*/*v* solvent system as follows. Stock solution of **3** ($2{\cdot}10^{-4}$ M) in DMSO was diluted with adequate volume of DMSO (to reach volume of 0.5 mL). This was followed by addition of proper amount of stock solution ($1{\cdot}10^{-5}$ M) of given cation, and finally H_2O was added to reach volume of the sample of 1 mL. Final concentration of **3** in each sample equaled $2{\cdot}10^{-7}$ M. Excitation wavelength (λ_{ex}) was 270 nm. Stern–Volmer plots were used for the evaluation of detection parameters of **3**, namely Stern–Volmer constants (K_{SV}) and limit of detection (LOD) values.[7–10] Data for the calculations were collected for emission wavelength (λ_{em}) of 356 nm. K_{SV} values were taken as a slope value of *1*/C($_{cation}$) *versus* 1/Δ*I* linear regression plots. LOD values were estimated from the intercept and slope of the linear regression plots of $(I-I_{min})/(I_{max}-I_{min})$ *versus* $\log(C_{cation})$ as follows. At first, the x(y=1) value was calculated. Next, LOD was taken as $10^{x(y=1)}$. Spectrofluorimetric studies on systems' stoichiometries (continuous variation method, Job's plot method[11]) were performed in DMSO/H_2O = 1:1 *v*/*v* solvent system with the samples comprising total number of moles of the receptor (compound **3**) and analyte (cations) of $4{\cdot}10^{-8}$ mol. Samples varied in the molar fraction (x_{cation}) of the analyte, ranging from 0.00 to 0.94.

S2. Synthesis

5'-(4-(Di(prop-2-yn-1-yl)amino)phenyl)-*N*4,*N*4,*N*4'',*N*4''-tetra(prop-2-yn-1-yl)-[1,1':3',1''-terphenyl]-4,4''-diamine 2. A mixture of amine **1** (0.28 mmol, 100 mg), propargyl bromide (288 μL of 80% solution in toluene), potassium carbonate (3.15 mmol, 446 mg) and acetonitrile (5 mL) was refluxed for 48 hours and filtered through a Celite® pad. The pad was washed with acetonitrile (15 ml). Volatiles were distilled off from the collected filtrates under reduced pressure (rotary evaporator). The residue was triturated with diethyl ether (2 mL) and filtered to yield compound **2** (157 mg, 97%) as yellow glassy solid. ^{1}H NMR (500 MHz, Chloroform-d) δ 7.72 (s, 3H), 7.68 (d, $^{3}J_{HH}$ = 8.8 Hz, 6H), 7.10 (d, $^{3}J_{HH}$ = 8.8 Hz, 6H), 4.22 (d, $^{4}J_{HH}$ = 2.5 Hz, 12H), 2.34 (t, $^{4}J_{HH}$ = 2.4 Hz, 6H). ^{13}C{^{1}H} NMR (126 MHz, Chloroform-d) δ 147.06, 141.70, 132.65, 128.03, 123.35, 115.81, 79.26, 72.95, 40.49. APCI-HRMS (q-TOF) m/z calcd. for $C_{42}H_{34}N_{3}$ $[M+H]^{+}$ 580.2753; found 580.2758.

5'-(4-(Bis((1-(ferrocenylmethyl)-1*H*-1,2,3-triazol-4-yl)methyl)amino)phenyl)-*N*4,*N*4,*N*4'',*N*4''-tetrakis((1-(ferrocenylmethyl)-1*H*-1,2,3-triazol-4-yl)methyl)-[1,1':3',1''-terphenyl]-4,4''-diamine 3. A mixture of compound **2** (0.05 mmol, 27 mg), (ferrocenylmethyl)azide (0.28 mmol, 67 mg), copper(II) acetate (0.02 mmol, 4 mg), sodium ascorbate (0.03 mmol, 5 mg), water (0.5 mL) and *t*-butanol (0.5 mL) was stirred at room temperature for 5 days and volatiles were distilled off under reduced pressure (rotary evaporator). The residue was dissolved in ethyl acetate (25 mL) and filtered through a Celite® pad. The pad was washed with methanol (10 ml). Volatiles were distilled off from the collected filtrates under reduced pressure (rotary evaporator). The residue was dissolved in DMSO (50 mL) and filtered through a Celite® pad. The pad was washed with DMSO (2 ml). The filtrate was lyophilized to yield compound **3** as brown amorphous solid (42 mg, 42%). ^{1}H NMR (500 MHz, DMSO-d_6) δ 7.97 (s, 6H), 7.50 (d, $^{3}J_{HH}$ = 8.5 Hz, 6H), 6.95 (d, $^{3}J_{HH}$ = 8.5 Hz, 6H), 5.26 (s, 12H), 4.68 (s, 12H), 4.29 (t, $^{3}J_{HH}$ = 12 Hz, 2H), 4.17 – 4.12 (m, 42H). ^{1}H DOSY NMR (500 MHz, DMSO-d_6), D $9.08 \cdot 10^{-11}$ m^2/s. Due to the very low solubility of **3** in common NMR solvents, no meaningful ^{13}C{^{1}H} NMR spectrum could be obtained. Purity of compound **3** (P_{Sample}, 98.34%) as mass fraction (%) was calculated from qNMR experiment with dimethyl sulfone, the certified reference material (CRM, Sigma-Adrich, product no. 41867); see **Figure S4**. The formula applied for the calculation of the sample purity was taken from the Certificate of Analysis of the CRM. Mass of sample, m_{Sample} = 0.531 mg. Mass of CRM, m_{CRM} = 0.064 mg. m_{CRM} was calculated taking into account the exact mass of the CRM in a 1.71 mg/mL stock solution and the dilution factor during sample preparation (×1/10). Purity of the CRM, P_{CRM} = 99.82%. Integral of the analyte signal (4.665 ppm), $I_{Analyte}$= 12.22. $I_{Analyte}$ was calculated as the average of signals observed at 4.12–7.97 ppm. Integral of the CRM signal (2.976 ppm), I_{CRM} = 16.09. Number of the analyte nuclei, $N_{Analyte}$ = 12. Number of the CRM nuclei, N_{CRM} = 6. Molecular mass of the analyte, $M_{Analyte}$ = 2026.20 g/mol. Molecular mass of the CRM, M_{CRM} = 94.13 g/mol. ESI-HRMS (TOF) m/2z calcd. for $C_{108}H_{100}Fe_{6}N_{21}$ $[M-H]^{+}$ 1013.22781; found 1013.22508 [relative abundance: 1011.72491 (8%), 1012.22721 (32%), 1012.72435 (57%), 1013.22508 (94%), 1013.72702 (100%), 1014.2288g (60%), 1014.73140 (23%), 1015.2320g (5%)].

S3. Spectral characterization data for 2-3

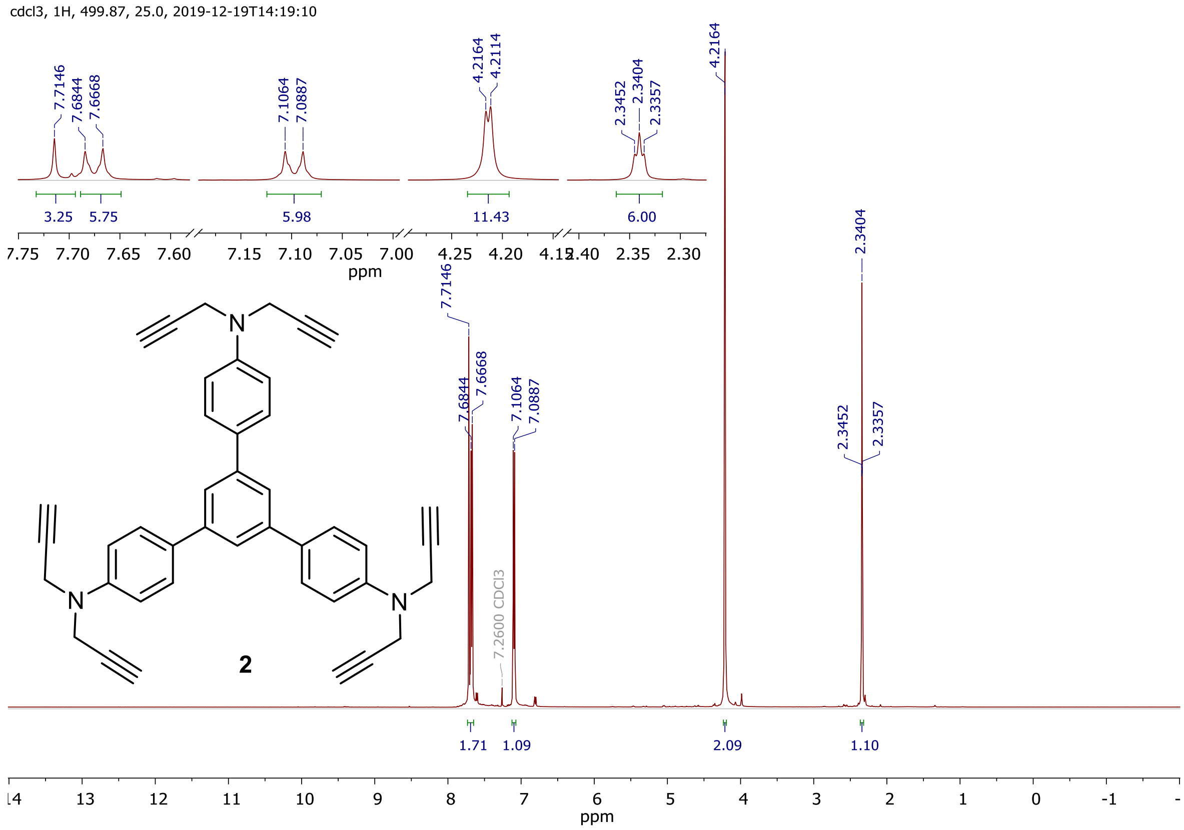

Figure S1. ^{1}H NMR spectrum of compound **2** (500 MHz, Chloroform-d).

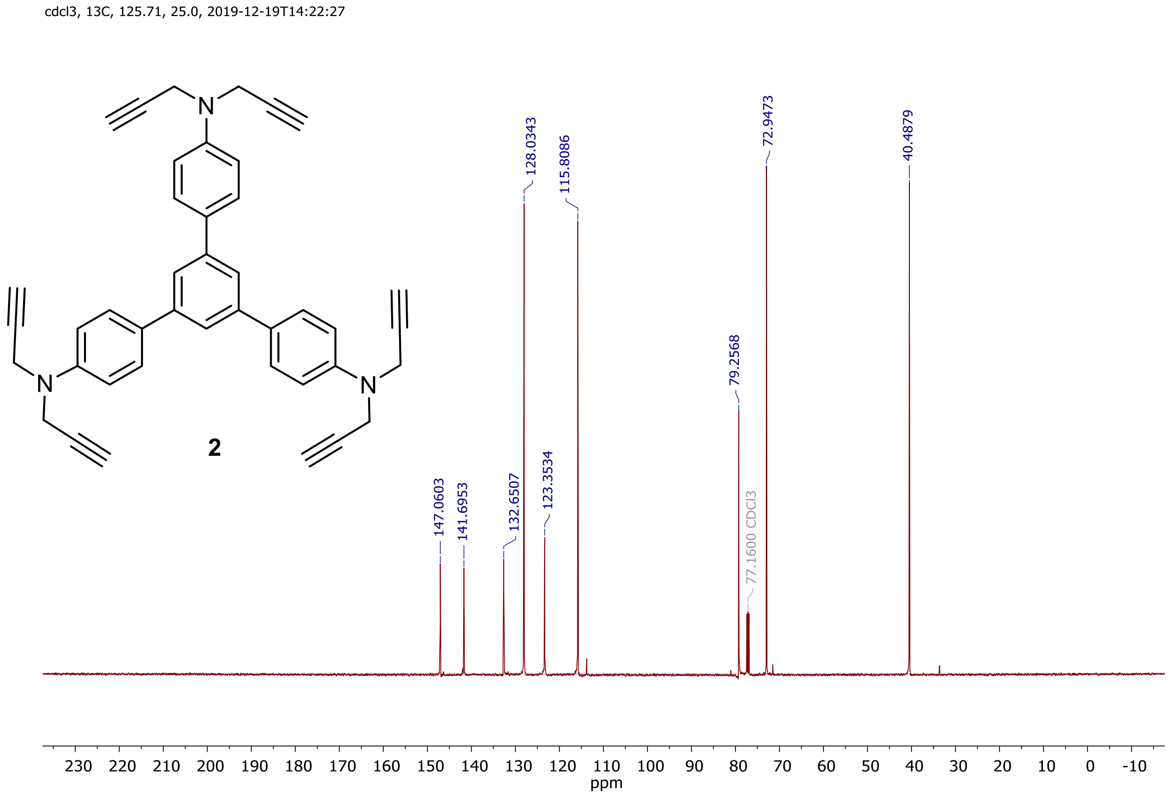

Figure S2. ^{13}C NMR spectrum of compound **2** (126 MHz, Chloroform-d).

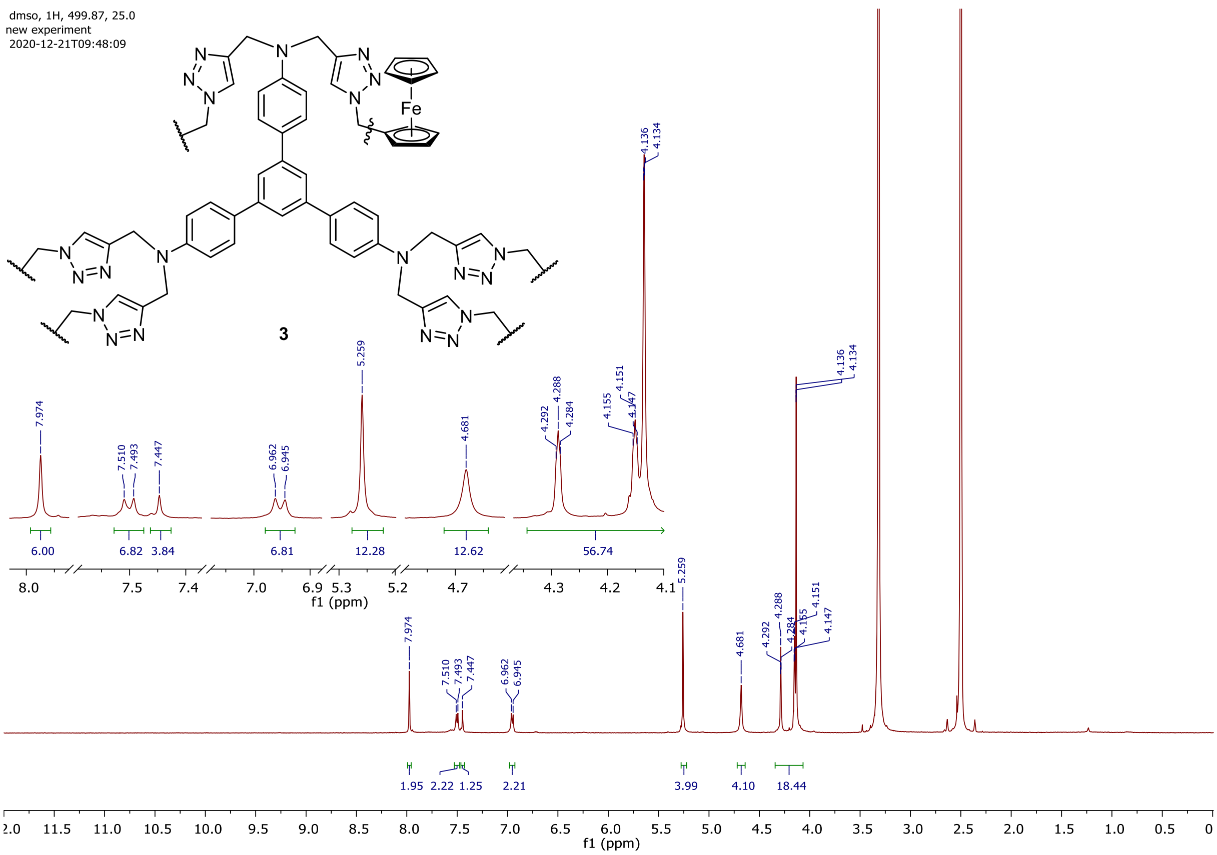

Figure S3. ^{1}H NMR spectrum of compound **3** (500 MHz, DMSO-d_6).

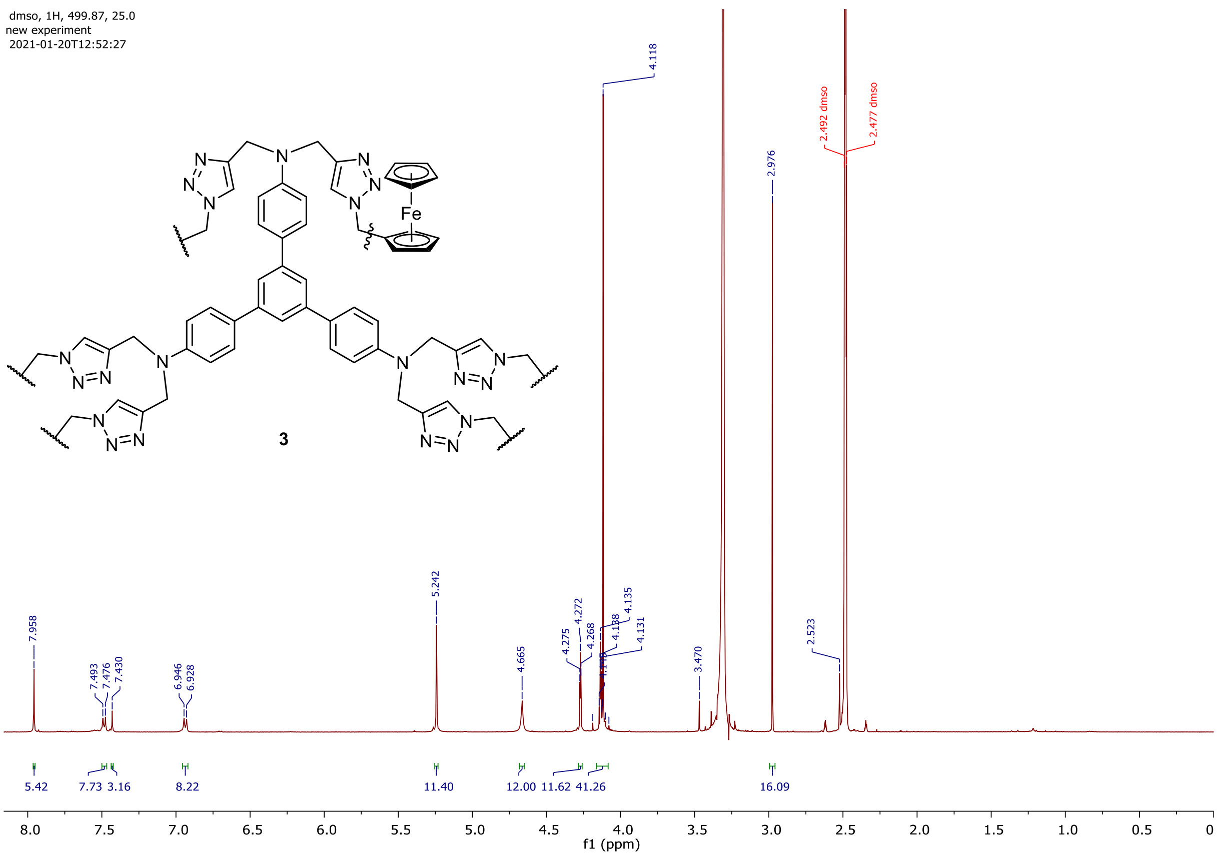

Figure S4. ^{1}H NMR spectrum of compound **3** (500 MHz, DMSO-d_6) for the purpose of qNMR analysis.

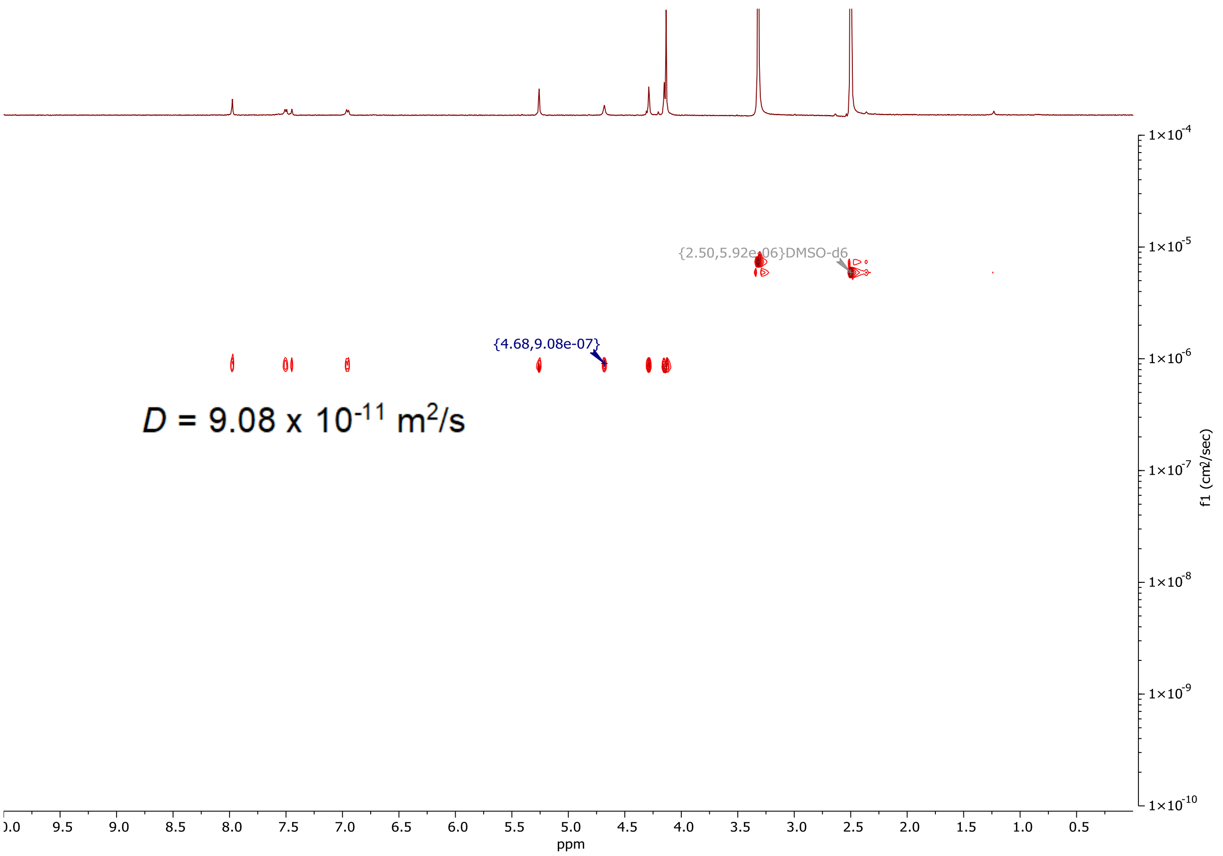

Figure S5. ^{1}H DOSY NMR spectrum of compound **3** (500 MHz, DMSO-d_6).

Single Mass Analysis
Tolerance = 3.0 mDa / DBE: min = -1.5, max = 300.0
Element prediction: Off
Number of isotope peaks used for i-FIT = 3
Monoisotopic Mass, Even Electron Ions
23 formula(e) evaluated with 1 results within limits (up to 50 closest results for each mass)
Elements Used:
C: 0-120 H: 0-200 N: 1-3

Mass	Calc. Mass	mDa	PPM	DBE	Formula	i-FIT	i-FIT Norm	Fit Conf %	C	H	N
580.2758	580.2753	0.5	0.9	27.5	C42 H34 N3	698.5	n/a	n/a	42	34	3

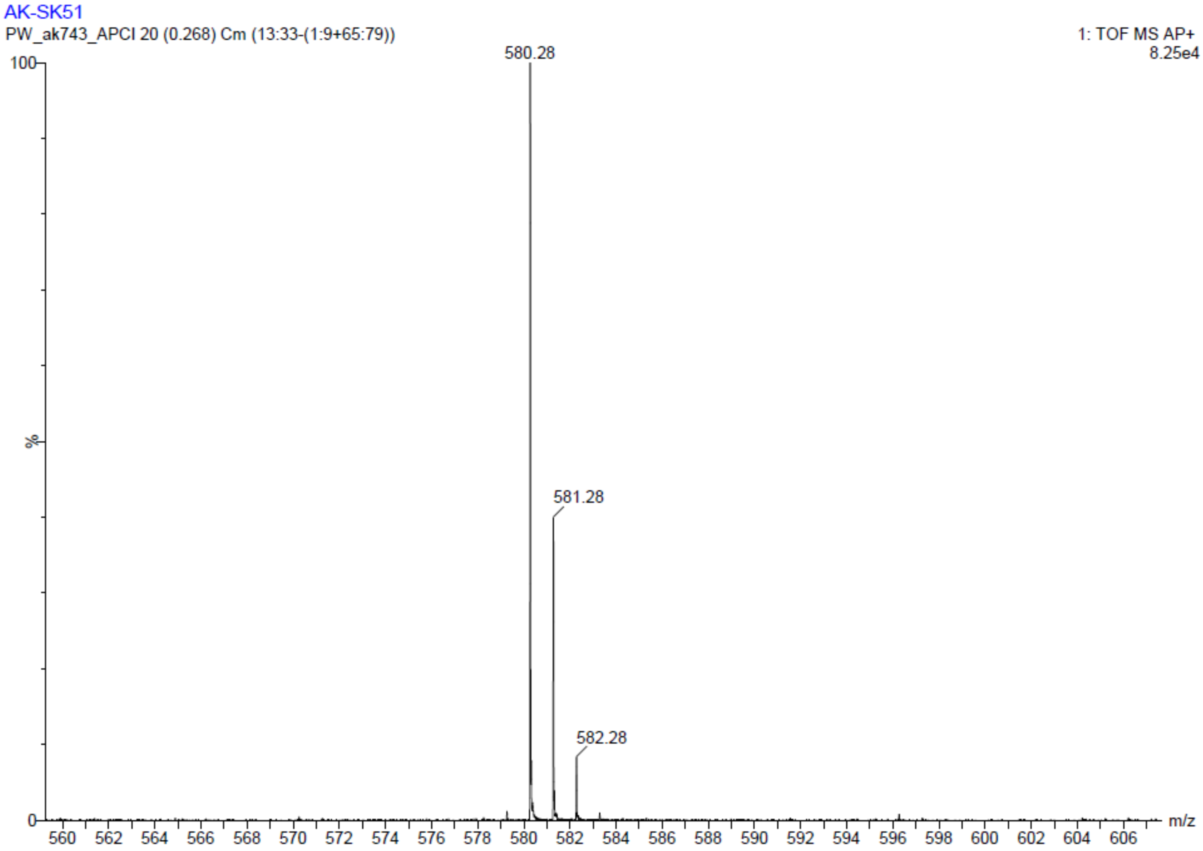

Figure S6. APCI-HRMS (q-TOF) spectrum of compound **2**.

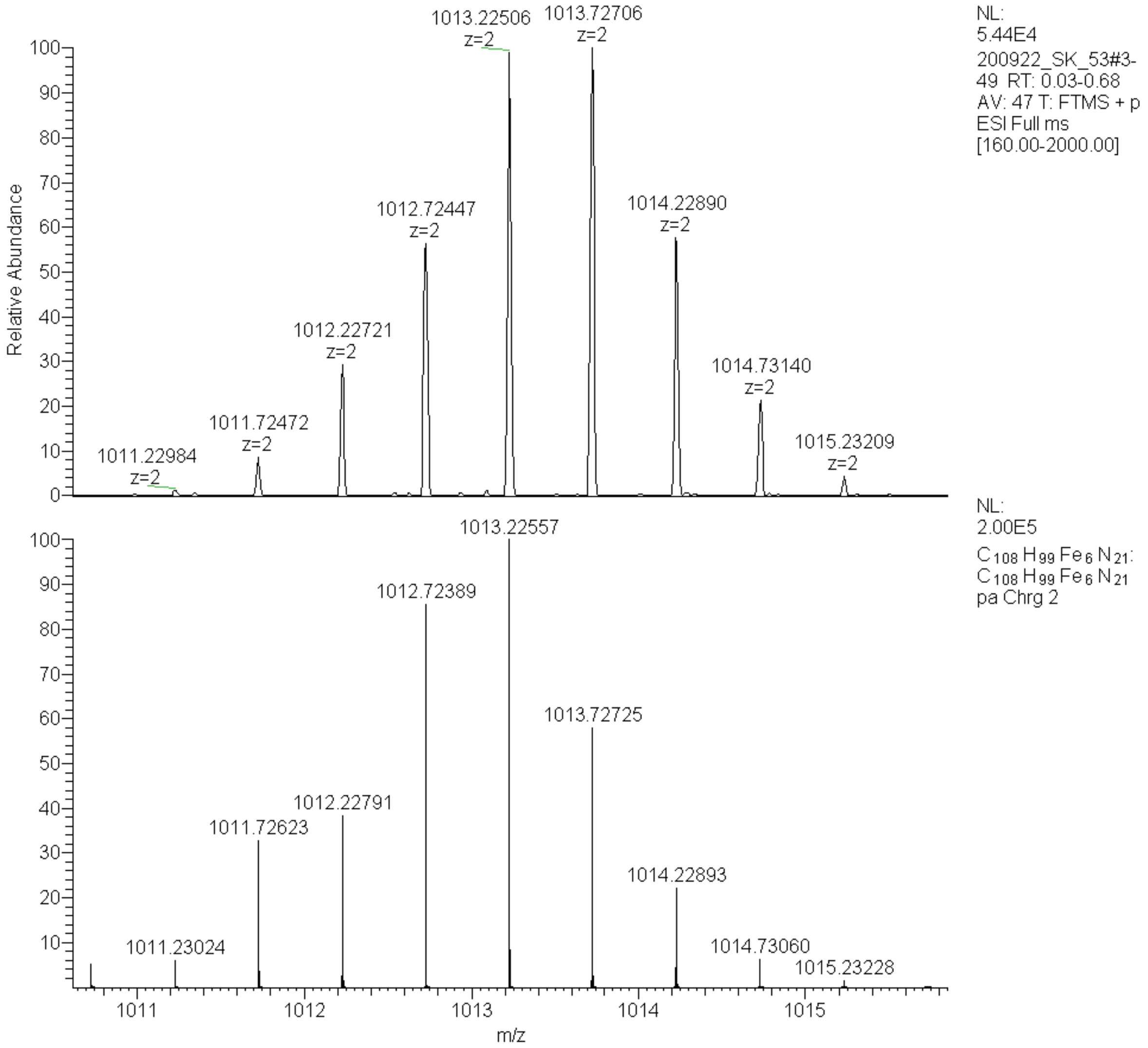

Figure S7. ESI-HRMS (TOF) spectrum of compound **3**.

S4. Electrochemical analyzes

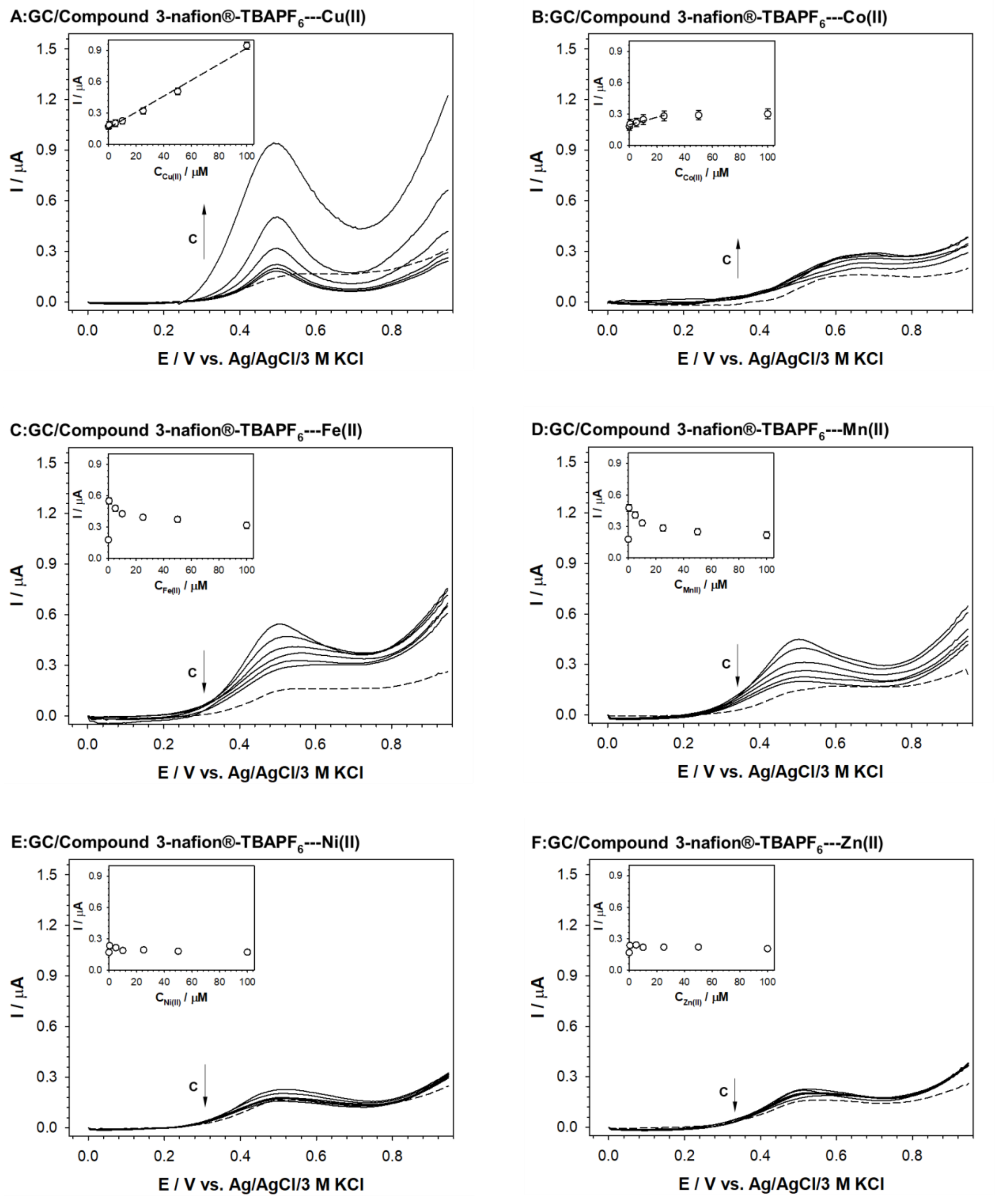

Figure S8. DP voltammograms of the receptor (GC/compound **3**-nafion-TBAPF$_6$) recorded in 100 mM TBABF$_4$ aqueous solution (dashed lines) with different addition of selected divalent transition metals (solid lines). Insets: Dependencies of anodic peak currents *vs.* divalent transition cation concentration. Experimental conditions: C_3 = 1.7 mM, T = 21 °C, modulation time: 0.002 s, interval time: 0.1 s, modulation amplitude: 0.04995 V.

S5. Stern-Volmer plots for the interactions between 3 and metal cations

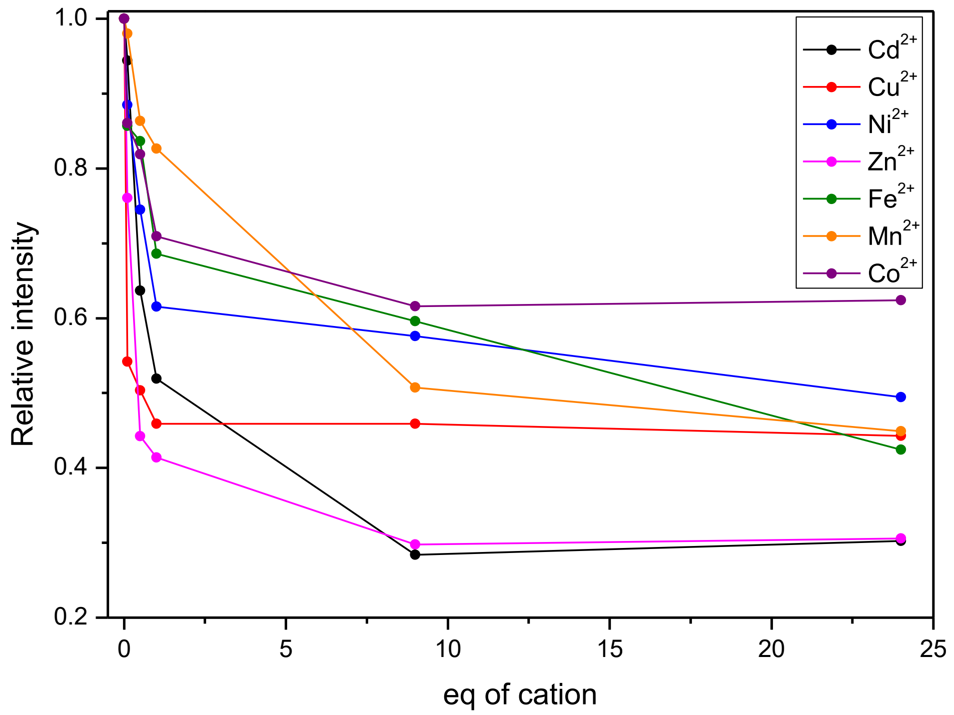

Figure S9. Relative changes in the emission intensity of compound **3** in the presence of increasing molar equivalents of given transition metal cation in solution ($C_3 = 2 \times 10^{-7}$ M, DMSO:H_2O 1:1 *v*/*v*, $\lambda_{ex} = 270$ nm).

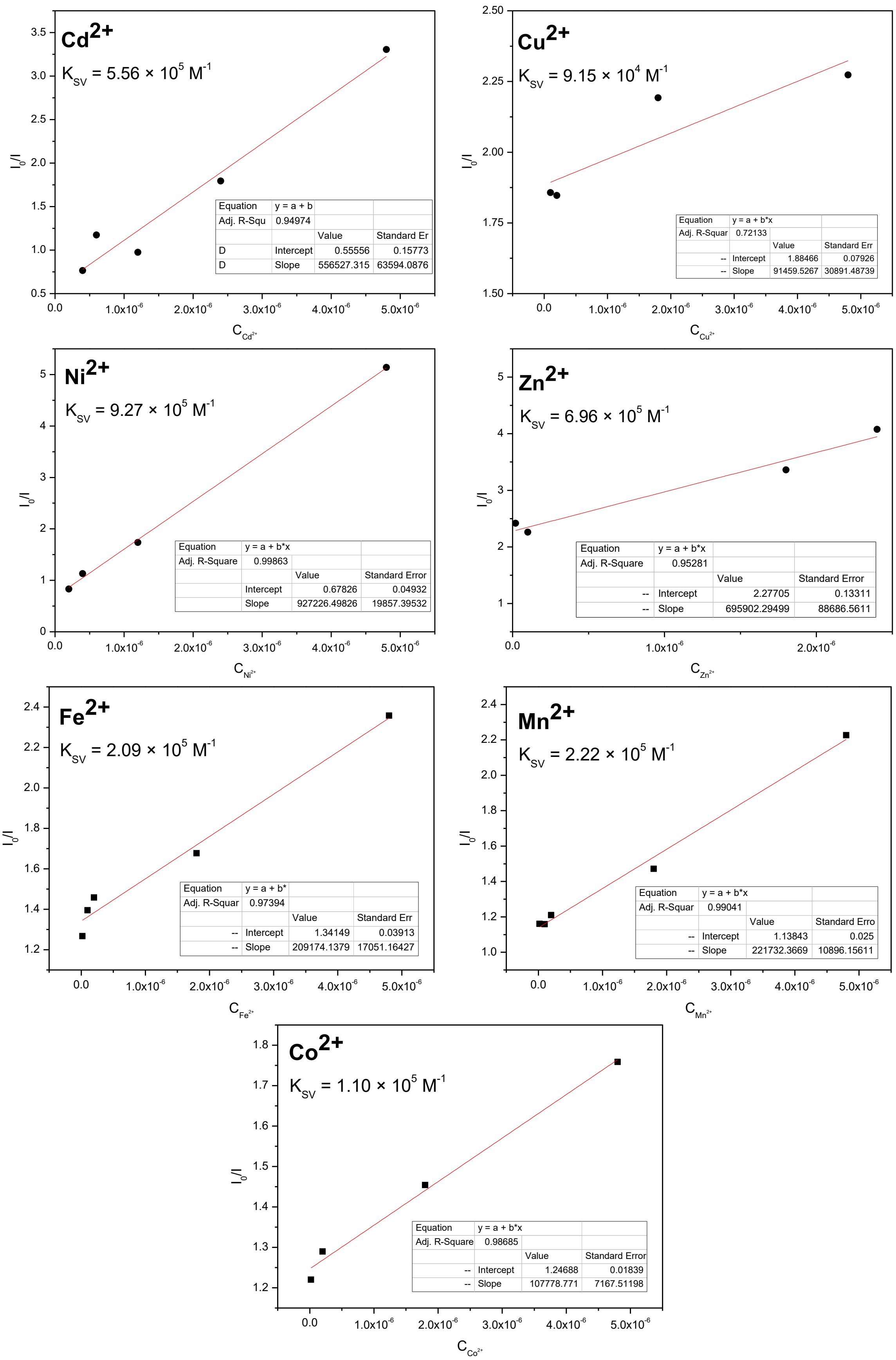

Figure S10. Stern-Volmer plots for the emission quenching of **3** by the addition of metal cations. Parameters of the linear regression, as well as calculated Stern-Volmer constant (K_{SV}) values are also provided.

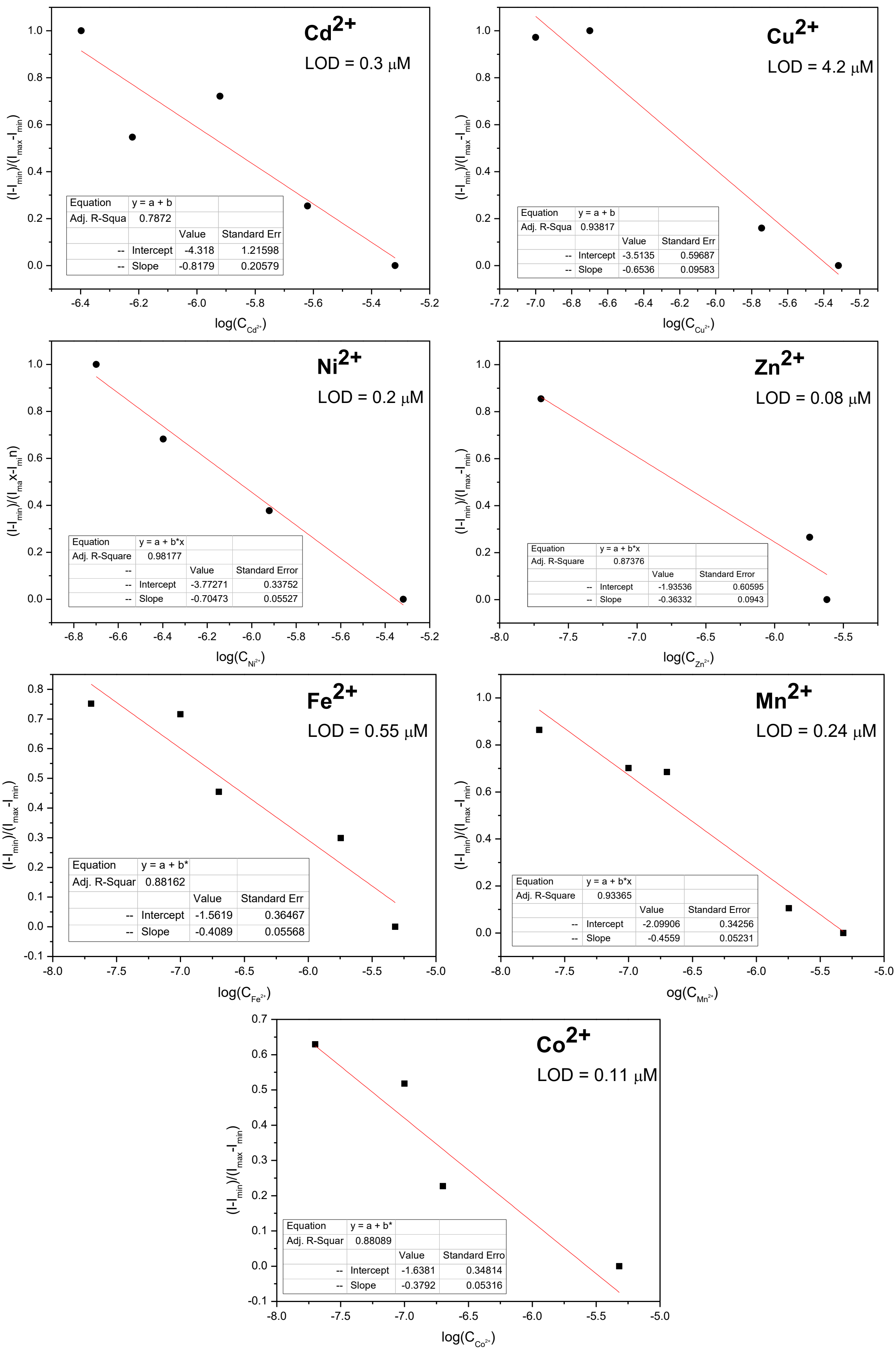

Figure S11. Plots of $(I{-}I_{min})/(I_{max}{-}I_{min})$ vs $\log(C_{cation})$ for the estimation of limit of detection (LOD) values. Parameters of the linear regression, as well as calculated LOD values are also provided.

S6. Job's plots for the interactions between 3 and metal cations

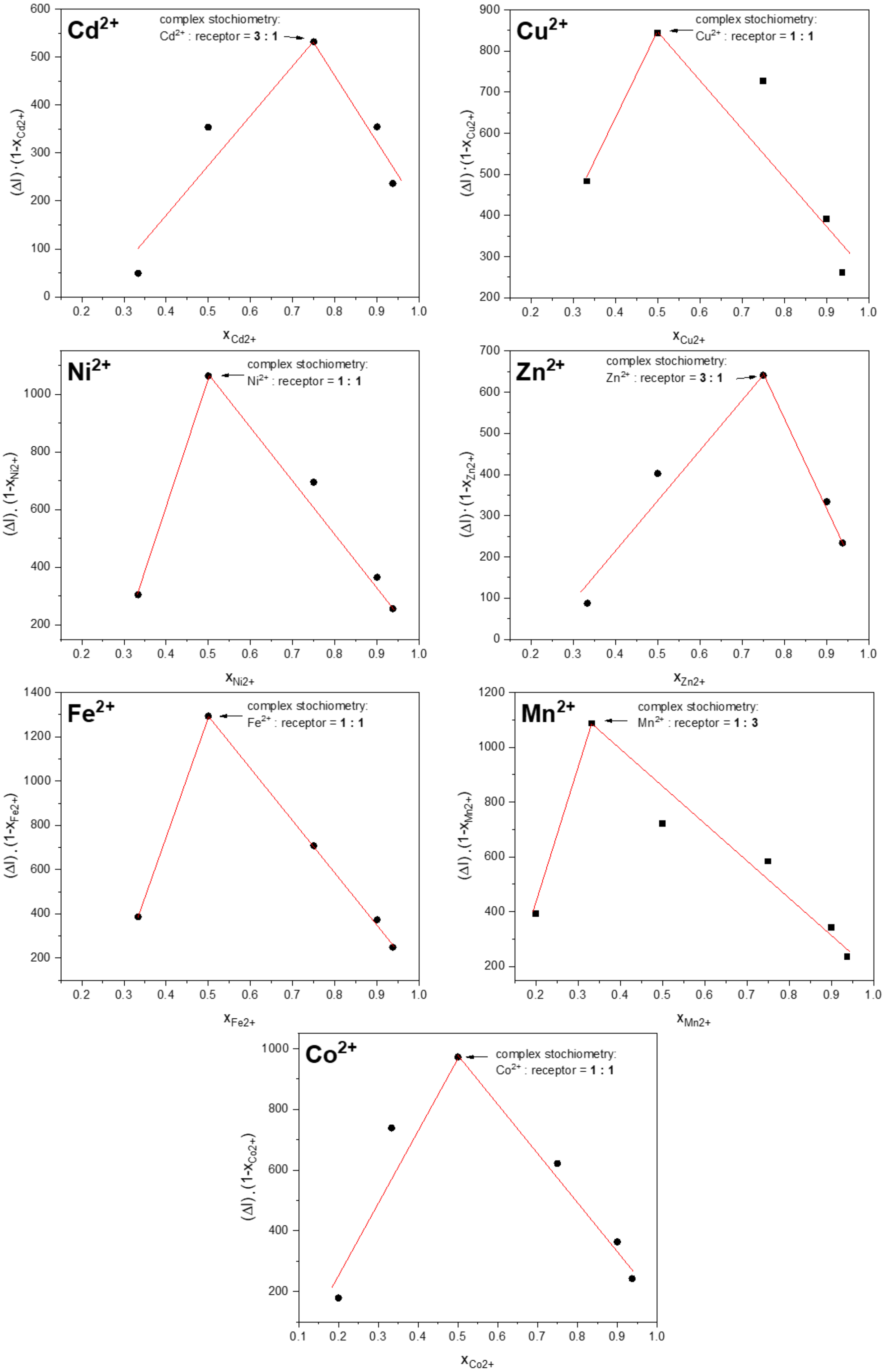

Figure S12. Job's plots for interactions between **3** and metal cations.

S7. DFT calculations methodology

Theoretical calculations were performed using Orca 5.0 program.[12] Molecules were optimized using BP86 method[13] with def2-TZVP basis set[14] augmented with def2/J auxiliary basis set[15] with RI approximation[16]. The calculations were performed in the presence of the solvent field with the polarizable continuum model (PCM) using the CPCM polarizable conductor calculation model.[17] The parameters of water were used. The starting geometries were constructed using Avogadro program.[18] Following geometry optimization, the vibrational frequencies were calculated, and the results showed that optimized structures are stable geometric structures (no imaginary frequencies). Gibbs free energies at 25 °C were obtained from the frequency calculations.

The calculations were performed for the following molecules: a) a single water molecule, b) Cd^{2+} ion hydrated by six water molecules, c) three chosen representative fragments of the receptor, d) complexes of Cd^{2+} with the chosen fragments of the receptor and water molecules. The initial geometries of the complexes were constructed in such a way that Cd^{2+} would adopt octahedral geometry with either receptor atoms and water molecules as ligands. The binding energies of the complexes were calculated from the equation:

$$\Delta G_{bind} = \Delta G_{complex} + (6 - n) \cdot \Delta G_{water} - \Delta G_{receptor} - \Delta G_{ion}$$

Where: ΔG_{bind} - free energy of the binding, $\Delta G_{complex}$ - free energy of the complex, n - number of water molecules included in the complex, ΔG_{water} - free energy of a single water molecule, ΔG_{ligand} - free energy of the receptor fragment, ΔG_{ion} - free energy of Cd^{2+} hydrated by six water molecules.

S8. Supporting references

(1) Xu, W.; Fan, Y.; Wang, H.; Teng, J.; Li, Y.; Chen, C.; Fenske, D.; Jiang, J.; Su, C. Investigation of Binding Behavior between Drug Molecule 5-Fluoracil and M_4L_4-Type Tetrahedral Cages: Selectivity, Capture, and Release. *Chem. Eur. J.* **2017**, *23* (15), 3542–3547. https://doi.org/10.1002/chem.201606060.

(2) Casas-Solvas, J. M.; Ortiz-Salmerón, E.; Giménez-Martínez, J. J.; García-Fuentes, L.; Capitán-Vallvey, L. F.; Santoyo-González, F.; Vargas-Berenguel, A. Ferrocene-Carbohydrate Conjugates as Electrochemical Probes for Molecular Recognition Studies. *Chem. Eur. J.* **2009**, *15* (3), 710–725. https://doi.org/10.1002/chem.200800927.

(3) Wu, D. H.; Chen, A. D.; Johnson, C. S. An Improved Diffusion-Ordered Spectroscopy Experiment Incorporating Bipolar-Gradient Pulses. *J. Magn. Res. A* **1995**, *115* (2), 260–264. https://doi.org/10.1006/jmra.1995.1176.

(4) Jerschow, A.; Müller, N. Suppression of Convection Artifacts in Stimulated-Echo Diffusion Experiments. Double-Stimulated-Echo Experiments. *J. Magn. Res.* **1997**, *125* (2), 372–375. https://doi.org/10.1006/jmre.1997.1123.

(5) Viel, S.; Ziarelli, F.; Pagès, G.; Carrara, C.; Caldarelli, S. Pulsed Field Gradient Magic Angle Spinning NMR Self-Diffusion Measurements in Liquids. *J. Magn. Res.* **2008**, *190* (1), 113–123. https://doi.org/10.1016/j.jmr.2007.10.010.

(6) Kunde, T.; Nieland, E.; Schröder, H. V.; Schalley, C. A.; Schmidt, B. M. A Porous Fluorinated Organic [4+4] Imine Cage Showing CO_2 and H_2 Adsorption. *Chem. Commun.* **2020**, *56* (35), 4761–4764. https://doi.org/10.1039/D0CC01872D.

(7) *Principles of Fluorescence Spectroscopy*; Lakowicz, J. R., Ed.; Springer US: Boston, MA, 2006. https://doi.org/10.1007/978-0-387-46312-4.

(8) Maurya, N.; Bhardwaj, S.; Singh, A. K. Selective Colorimetric and Fluorescence ‘Turn-on’ Sensor for Ag^+ and in-Situ Sensing of CN^- (off–on-off) via Displacement Approach. *Mat. Sci. Eng. C* **2017**, *74*, 55–61. https://doi.org/10.1016/j.msec.2016.12.131.

(9) Han, J.; Yakiyama, Y.; Takeda, Y.; Sakurai, H. Sumanene-Functionalised Bis(Terpyridine)–Ruthenium(II) Complexes Showing Photoinduced Structural Change and Cation Sensing. *Inorg. Chem. Front.* **2023**, *10* (1), 211–217. https://doi.org/10.1039/D2QI01801B.

(10) Ding, Y.; Xie, Y.; Li, X.; Hill, J. P.; Zhang, W.; Zhu, W. Selective and Sensitive “Turn-on” Fluorescent Zn^{2+} Sensors Based on Di- and Tripyrrins with Readily Modulated Emission Wavelengths. *Chem. Commun.* **2011**, *47* (19), 5431–5433. https://doi.org/10.1039/C1CC11493J.

(11) Renny, J. S.; Tomasevich, L. L.; Tallmadge, E. H.; Collum, D. B. Method of Continuous Variations: Applications of Job Plots to the Study of Molecular Associations in Organometallic Chemistry. *Angew. Chem. Int. Ed.* **2013**, *52* (46), 11998–12013. https://doi.org/10.1002/anie.201304157.

(12) Neese, F.; Wennmohs, F.; Becker, U.; Riplinger, C. The ORCA Quantum Chemistry Program Package. *J. Chem. Phys.* **2020**, *152* (22), 224108. https://doi.org/10.1063/5.0004608.

(13) Becke, A. D. Density-Functional Exchange-Energy Approximation with Correct Asymptotic Behavior. *Phys. Rev. A* **1988**, *38* (6), 3098–3100. https://doi.org/10.1103/PhysRevA.38.3098.

(14) Weigend, F.; Ahlrichs, R. Balanced Basis Sets of Split Valence, Triple Zeta Valence and Quadruple Zeta Valence Quality for H to Rn: Design and Assessment of Accuracy. *Phys. Chem. Chem. Phys.* **2005**, *7* (18), 3297. https://doi.org/10.1039/b508541a.

(15) Weigend, F. Accurate Coulomb-Fitting Basis Sets for H to Rn. *Phys. Chem. Chem. Phys.* **2006**, *8* (9), 1057. https://doi.org/10.1039/b515623h.

(16) Dunlap, B. I.; Connolly, J. W. D.; Sabin, J. R. On Some Approximations in Applications of *X* α Theory. *J. Chem. Phys.* **1979**, *71* (8), 3396–3402. https://doi.org/10.1063/1.438728.

(17) Tomasi, J.; Mennucci, B.; Cammi, R. Quantum Mechanical Continuum Solvation Models. *Chem. Rev.* **2005**, *105* (8), 2999–3094. https://doi.org/10.1021/cr9904009.

(18) Hanwell, M. D.; Curtis, D. E.; Lonie, D. C.; Vandermeersch, T.; Zurek, E.; Hutchison, G. R. Avogadro: An Advanced Semantic Chemical Editor, Visualization, and Analysis Platform. *J. Cheminform.* **2012**, *4* (1), 17. https://doi.org/10.1186/1758-2946-4-17.
